# Construction of a high density SNP linkage map of kelp (*Saccharina japonica*) by sequencing *Taq* I site associated DNA and mapping of a sex determining locus

**DOI:** 10.1186/s12864-015-1371-1

**Published:** 2015-03-15

**Authors:** Ning Zhang, Linan Zhang, Ye Tao, Li Guo, Juan Sun, Xia Li, Nan Zhao, Jie Peng, Xiaojie Li, Liang Zeng, Jinsa Chen, Guanpin Yang

**Affiliations:** Laboratory of Marine Genetics and Breeding, Ocean University of China, Qingdao, 266003 China; Institute of Evolution and Marine Biodiversity, Ocean University of China, Qingdao, 266003 China; College of Marine Life Sciences, Ocean University of China, Qingdao, 266003 China; National Engineering Science Research & Development Center of Algae and Sea Cucumbers of China; Provincial Key Laboratory of Genetic Improvement & Efficient Culture of Marine Algae of Shandong, Shandong Oriental Ocean Sci-tech Co., Ltd, Yantai, Shandong 264003 China; Majorbio Pharm Technology Co., Ltd, Shanghai, 201203 China

**Keywords:** Kelp, *Saccharina japonica*, SNP, RAD, Linkage map, Sex determining locus

## Abstract

**Background:**

Kelp (*Saccharina japonica*) has been intensively cultured in China for almost a century. Its genetic improvement is comparable with that of rice. However, the development of its molecular tools is extremely limited, thus its genes, genetics and genomics. Kelp performs an alternative life cycle during which sporophyte generation alternates with gametophyte generation. The gametophytes of kelp can be cloned and crossed. Due to these characteristics, kelp may serve as a reference for the biological and genetic studies of *Volvox*, mosses and ferns.

**Results:**

We constructed a high density single nucleotide polymorphism (SNP) linkage map for kelp by restriction site associated DNA (RAD) sequencing. In total, 4,994 SNP-containing physical (tag-defined) RAD loci were mapped on 31 linkage groups. The map expanded a total genetic distance of 1,782.75 cM, covering 98.66% of the expected (1,806.94 cM). The length of RAD tags (85 bp) was extended to 400–500 bp with Miseq method, offering us an easiness of developing SNP chips and shifting SNP genotyping to a high throughput track. The number of linkage groups was in accordance with the documented with cytological methods. In addition, we identified a set of microsatellites (99 in total) from the extended RAD tags. A gametophyte sex determining locus was mapped on linkage group 2 in a window about 9.0 cM in width, which was 2.66 cM up to marker_40567 and 6.42 cM down to marker_23595.

**Conclusions:**

A high density SNP linkage map was constructed for kelp, an intensively cultured brown alga in China. The RAD tags were also extended so that a SNP chip could be developed. In addition, a set of microsatellites were identified among mapped loci, and a gametophyte sex determining locus was mapped. This map will facilitate the genetic studies of kelp including for example the evaluation of germplasm and the decipherment of the genetic bases of economic traits.

**Electronic supplementary material:**

The online version of this article (doi:10.1186/s12864-015-1371-1) contains supplementary material, which is available to authorized users.

## Background

In Chinese aquaculture community, *Saccharina japonica* is referred to as kelp [1,2] although other species, *e.g.*, giant kelp (*Macrocystis pyrifera*), have also been tentatively cultured in recent years. Chinese kelp breeders and farmers also call the cultured kelp either Japanese kelp or true kelp as its scientific name *S. japonica* indicated; unfortunately the cultured kelp of China may have been contaminated genetically by *S. longissima* as two interspecific hybrids [3,4] and a hybrid-derived variety [5] at least have been developed and commercially cultured recently. Kelp has contributed significantly to Chinese mariculture. Its culturing area reached 40,201 hectares and its yield (weight of air dried frond) reached 979,006 tons in 2012 as was documented in Annual Report of Chinese Fisheries 2013. Kelp can be used to extracting mannitol, alginate and medicine or as human food and animal feed. Kelp promises also to be the most favorable feedstock for bioethanol fermentation as its three major components, mannitol, laminarian and alginate, can be fermented into bioethanol concertedly [6-8]. Kelp may also serve as the carbon fixers [9]. In addition, kelp has been integrated into environmental remediation and healthy animal culture systems in China.

China has cultured kelp intensively for almost a century. As were widely practiced in terrestrial crops, kelp has also been genetically improved with methods including continuous selection, hybridization and selection and development of hybrids. Since 1950s, more than 20 elite varieties and hybrids have been bred, which have contributed significantly to the culture performance of kelp, especially its yield. The representatives of these varieties included Haiqin no.1 [10], 901 [5] and Dongfang no.2 [4]. The weakness of their stress tolerance and their inapplicability for processing may not satisfy the tremendous and diverse requirements of kelp as human food, animal feed and industrial raw material; however the yield of these kelp varieties, especially that of hybrids, has been significantly elevated. Actually, the yield of hybrid kelps increases by 60-70% over normal varieties, almost a half of the theoretical, *i.e.* 59 metric tons/ha/year [11]. Such a margin of yield increase is more than that of hybrid rice over normal rice varieties (≥20%) [12]. Kelp is the only macroalga that has received systematic genetic improvement; unfortunately the studies on its genes, genetics and genomics are far behind those of rice.

Kelp performs an alternative life cycle during which sporophyte generation alternates with gametophyte generation [13]. The sporophytes are large and multicellular while the gametophytes are microscopic, containing a single or a few cells. The asexual sporophytes (diploid) produce motile zoospores (monoploid). These zoospores develop into sexual male and female gametophytes which produce spermatozoids and eggs, respectively. Through fertilization, spermatozoids and eggs fuse to form zygotes which subsequently develop into sporophytes. Kelp gametophytes can asexually propagate [14-16], forming gametophyte clones applicable to either germplasm conservation or gametophyte clone hybridization and hybrid kelp seedling raising [3-5]. This avoids the dependence of mature sporophytes met in traditional summer seedling-raising from sporophytes [17]. Year round seedling-raising of kelp is potent as the maturing time is different among habitats [18] and physiologically modifiable [19]. The field culturing facility of kelp in China has evolved from floating raft [13] to floating rope net [3]. The seedlings were placed in between the skeins of the pendent ropes between floating head-ropes fixed at two ends to sea bed. In addition, both kelp sporophytes and gametophytes have survived the genetic transformation with diverse methods [20]. The number of chromosomes in kelp nucleus is hard to determine as they are resistant to staining with traditional chemical dyes. Accordingly, the number of chromosomes in kelp nucleus has been debated for a long time. With an improved staining method, such a number was determined as 31 by Zhou *et al*. [21], which was further supported by 4, 6-diamino-2-phenyl indole (DAPI) staining [22-24]. Sex specific markers including inter-simple sequence repeat (SSR) [24] and a sequence [22] have been identified. Measurable and observable traits of kelp included at least spermatozoid life-span [25], temperature tolerance of young sporophytes [26], and those frequently evaluated during breeding, *e.g*. the morphological characteristics and stress tolerance [3-5]. These achievements make the intensive studies of kelp with molecular tools feasible and appreciable.

A SNP is a DNA sequence variation that occurs when a single nucleotide in the genome differs between either the members of a species or the paired chromosomes in a single individual. SNPs have gained a wide range of applications in humans and model species. They are becoming the marker of choice for additional species as well. The SNP genotyping techniques include tetra primer amplification refractory mutation system - polymerase chain reaction (T-ARMS-PCR) [27], capillary electrophoresis [28], high resolution melting [29], mass spectrometry [30,31], optical amplification of cationic conjugated polymers and the single base primer extension reaction [32], DNA microarray [33-36], pyrosequencing [37,38], multiplex PCR [39]; PCR and sequencing [40], competitive allele specific PCR (KASPar) [41], GoldenGate [42-45], resequencing [46], and among others [47]. This evolution has witnessed a dramatic increase in genotyping throughput; however the high cost, labor intensiveness and confirmation bias, alone or in combination, have slowed their application to a wide range of species and individuals within a given population. SNP genotyping by traditional DNA sequencing originally promised a high throughput, but its high cost limited its applicability to only model or extremely important species [48]. With the coming of the next generation (massively parallel) sequencing era, the cost of genotyping by sequencing is being progressively reduced, making the diversity determining, map constructing and trait mapping by sequencing feasible in organisms with or without reference genome sequence [49]. Implementation of methodologies such as the reduction of genome complexity [50] and restriction-site associated DNA (RAD) sequencing [36,51-53] and a modification of this method, double digest RAD (ddRAD) [54-56] has dramatically widened the application of genotyping by sequencing in linkage map construction [57,58] and association analysis [59]. In contrast, trait mapping on the SNP linkage maps constructed by sequencing has been scarce [60]. In addition to map construction and trait mapping, SNPs in chip format have also been used to genomic breeding of rice [61] and characterization of genomic diversity of wheat [62].

For kelp (*S. japonica*), diverse molecular markers have been developed, which included amplified fragment length polymorphism (AFLP) [63-65], SSR [66-69] and among others. These markers have been used to quantitative trait locus (QTL) mapping [70], locating gametophyte sex determining locus [64], diversity determination [71,72] and heterosis prediction [73]. However, kelp is far behind model plants and crops in the number and type of molecular markers and the applying depth and width of these markers although the breeding strategy and achievement of kelp, especially hybrid breeding, are comparable with those of rice and its potential of being a source feedstock of bioethanol fermentation. It is clear that the genetic studies of kelp should shift onto a fast molecular marker track as other plants do. In this study, a high density SNP linkage map was constructed for kelp by sequencing *Taq* I associated DNA, aiming to provide kelp studies an important tool.

## Methods

### Construction of a gametophyte clone mapping panel

The parental female gametophyte clone was isolated from Dongfang no. 3, a kelp hybrid [3], in 2004 while the parental male gametophyte clone was isolated from Benniu, a *S. japonica* variety, bred by continuous selection in 2003. The sporophytes were raised from these parental gametophyte clones and cultured with the methods described early [4]. In Jul. 2011, three well developed mature sporophytes were selected with their heterozygosity judged by genotyping at nine microsatellites (*H1*, *H10*, *H123*, *D3*, *D5*, *D9*, *Zspj22*, *Zspj28* and *Zspj38*) [66,68,69]. Tissue blocks with sporangia were cut from sporophytes, two each, which were scratched with cotton balls and rinsed with sterilized seawater thoroughly, submerged in 1.5% KI for 5-10 min, and air-dried for 2-3 h. The blocks were submerged in sterilized seawater independently, allowing zoospores to release. The zoospores were allowed to sink and adhered onto the bottom surface of a glass dish and germinate there in a few days. The young gametophytes were picked up once their sex is distinguishable and inoculated into seawater for further growth as we did early [4]. Out of six tissue blocks, only one was selected for constructing the gametophyte clone mapping panel. The seawater used for sporophyte tissue rinsing, zoospore releasing and germinating, young gametophyte culturing and conserving and among others was filtrated through fabric ester microhole filtering films (WX-0.22 μM, MOSU Instruments CO., LTD, Shanghai, China) and autoclaved at 121°C for 30 min.

### DNA extraction

**Figure 1:**
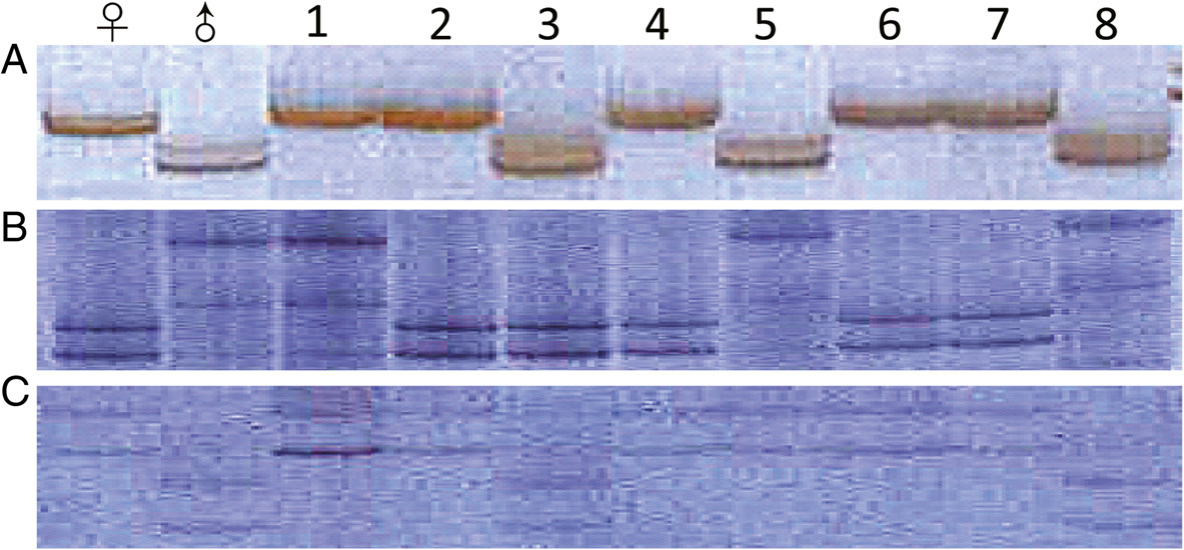
**Microsatellite segregation pattern of mapping panel.** Eight randomly selected gametophyte clones from mapping panel were genotyped with *D5* **(A)**; *H45* **(B)** and *H123* **(C)**. The gametophyte clones of mapping panel segregate as expected.

The genomic DNA of the gametophyte clones of mapping panel and 2 parental gametophyte clones was extracted primarily with a modified cetyltriethylammnonium bromide (CTAB) method [74]. About 1.0 g of gametophytes was ground into powder in liquid nitrogen. To the powder, 3 mL of extraction buffer (100 mM Tris–HCl pH 7.5, 50 mM ethylene diamine tetraacetic acid (EDTA) pH 4.5, 1.5 M NaCl, 2% CTAB, 1% (w/v) polyvinylpyrrolidone) was added. The mixture was shaken vigorously for 5 min and incubated at 55°C for 2 h in the presence of 20 units of proteinase K, and treated with 1/3 extraction buffer volume of 5 M KAc (pH 5.0) on ice for 30 min. The cellular debris and polysaccharides were removed by centrifuging at 12,000 rpm and 4°C for 15 min. The supernatant was extracted with an equal volume of phenol: chloroform: isoamyl alcohol (25:24:1 in volume) and then with an equal volume of chloroform: isoamyl alcohol (24:1) with polysaccharides removed again by adding 0.3 volumes of absolute ethanol and an equal volume of chloroform: isoamyl alcohol (24:1). The DNA was precipitated with 0.8 volume of cold isopropanol at −20°C for 1 h, washed twice with 70% cold ethanol and dissolved in 1 × TE. The DNA was further purified using Plant Genomic DNA Kit (Product No. DP305, Tiangen Biotech Co., Ltd, Beijing, China). The RNA was removed away with an appropriate amount of RNase. In case of biomass-limited, *e.g.* some male gametophyte clones; genomic DNA was extracted directly with Plant Genomic DNA Kit. In order to verify the segregation of the mapping panel, two parental gametophyte clones and eight randomly selected clones were subjected to microsatellite genotyping (Figure 1). The polymorphic microsatellites included *D5*, *H45* and *H123* [68,69]. The quality genomic DNA of the gametophyte clones of mapping panel was used for RAD sequencing.

### RAD sequencing and SNP linkage map construction

RAD library constructing, indexing and pooling were done with the strategy developed for natural populations [52]. The restriction endonuclease *Taq* I was used to cutting gametophyte clone DNA. A total of 24 multiplex sequencing libraries were constructed, in which each gametophyte clone DNA was prefixed with a unique nucleotide multiplex identifier as a barcode. *Taq* I end (101 bp in length) sequencing was performed on Illumina HiSeq2500 platform. Illumina Miseq PE250 was used to extending *Taq* I site associated DNA of two parental gametophyte clones. Raw RAD reads were trimmed to 85 nucleotide tags, which ensured > 97.5% of nucleotides have a quality value > Q30 (<0.1% sequencing error). These tags were aligned into *Taq* I site associated tag piles by their sequence similarity using Stacks [75]. Unique tags, *i.e.* the non-redundant with a maximum of one base difference from others, were screened out of a tag pile and used as the candidate alleles occupying a corresponding physical RAD locus. Physical RAD loci are sequence tagged while genetic loci are linkage determined. All candidate alleles were then collapsed into clusters using Stacks under default parameters for SNP calling [76]. Genotype calling, a process of determining the SNP genotype of physical RAD loci of each gametophyte clone after SNP calling, followed the philosophy of Hohenlohe *et al*. [76]. The customized perl scripts were applied then to generate a “.loc” file as the input of Joinmap 3.0 [77,78] with SNP linkage map calculated at a log of odds (LOD) value of 6.0 and a maximum recombination of 0.400 with regression algorithm. The linkage distances between loci were exported into MapChart [79] for map drawing.

The expected length of map (Ge) was the average of Ge1 and Ge2, where Ge1 was the sum of the lengths of all linkage groups, each revised by adding 2 s (s is the average space between loci, 2 s accounts for the two chromosome ends) to the observed [80], and Ge2 was the sum of the lengths of linkage groups, each revised by multiplying the observed with (m + 1)/(m - 1), where m is the number of genetic loci [81]. The genome coverage was calculated by dividing the observed map length with that of the expected as we did early [82].

## Results

### Gametophyte clone genotyping

**Figure 2:**
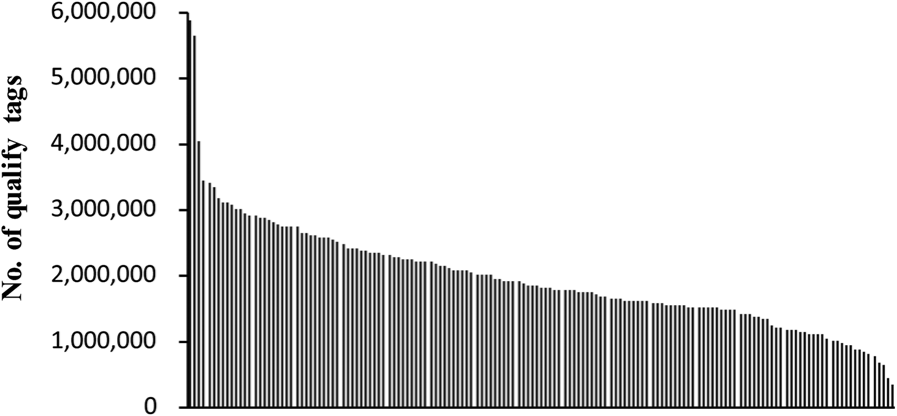
**The decreasing number of quality tags.** These tags were generated for parental female and male gametophyte clones (bar 1 and 2, respectively) and 140 gametophyte clones of mapping panel (bar 3 through 143).

Trimming the raw HiSeq2500 reads yielded the quality tags for gametophyte clones each, which ranged from 341,797 to 4,028,160 with an average of 1,905,908. For parental female and male gametophyte clones, 5,886,795 and 5,634,477 quality tags were generated, respectively (Figure 2). The quality tags of a gametophyte clone were aligned into RAD-tag piles with those covering only one tag discarded in order to ensure sequencing reliability. The remaining RAD-tag piles were considered as physical RAD loci from which unique candidate alleles were picked up. All candidate alleles identified among gametophyte clones of mapping panel were clustered for SNP and SNP genotype calling. Of 153 gametophyte clones, 14 were deleted in linkage map calculation as they were either genotype heterozygous or genotype absent at a large portion of physical RAD loci. The remaining 139 gametophyte clones were used for map calculation.

### Construction and characterization of SNP linkage map

**Figure 3:**
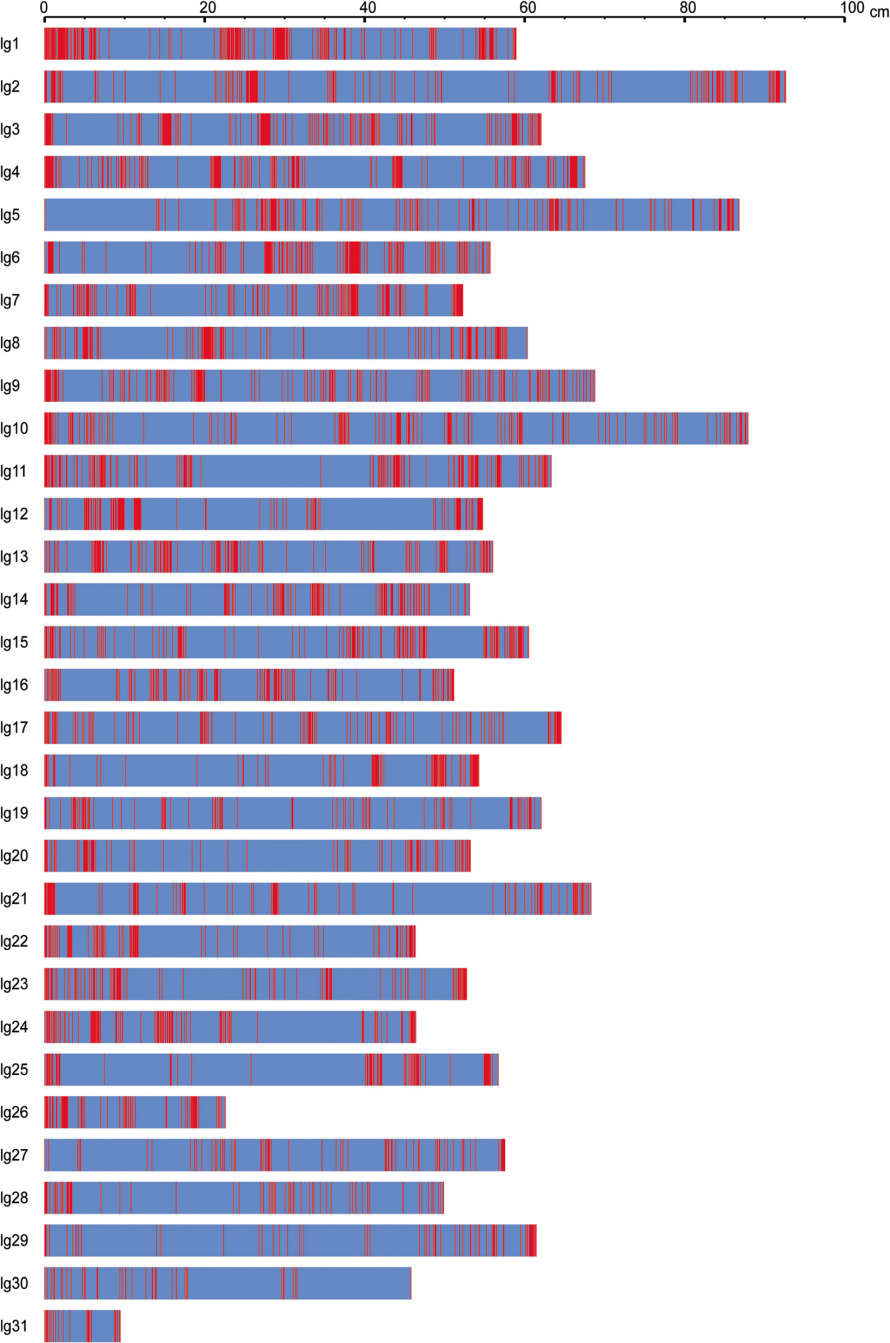
**A brief illustration of the linkage map.** The linkage map constructed was briefly shown. The illustrated include 31 linkage groups which are equal to the chromosomes reported early, and the unevenness of marker distribution on these groups. The horizontal bars represent linkage groups while the vertical (red) lines represent SNP markers.

In total, 4,994 physical RAD loci survived testing against 1:1 segregation expectation, grouping and map calculation, which were assigned onto 31 linkage groups finally (Figure 3, Additional files 1 and 2). These physical RAD loci occupied 4,921 genetic RAD loci as some of physical RAD loci co-segregated among gametophyte clones. The number of markers was large; however their distribution was not even (Figure 3). The maximum interval between genetic RAD loci was 14.97 cM while the minimum was 0.001 cM with an average of 0.36 cM (Table 1). Big intervals were often found in linkage groups each, indicating that some regions of kelp genome were extremely recombination less. The length of map was 1,782.75 cM in length, which accounted for 98.66% of the expected (1,806.94 cM).

**Table 1:** **The characteristics of the linkage map constructed in this study**

**LG**	**No. of**		**Space (cM)**			**Length (cM)**		
	**Markers**	**Loci**	**Max.**	**Min.**	**Avr.**	**Cal.**	**Rev.1**	**Rev.2**
1	308	304	5.123	0.001	0.195	58.913	59.638	59.303
2	279	262	9.846	0.001	0.356	92.619	93.344	93.331
3	278	276	6.530	0.001	0.226	62.056	62.781	62.509
4	268	268	8.258	0.001	0.254	67.532	68.257	68.040
5	252	249	13.957	0.001	0.351	86.804	87.529	87.507
6	227	225	5.008	0.001	0.250	55.674	56.399	56.173
7	220	216	6.874	0.001	0.244	52.262	52.987	52.750
8	217	211	8.349	0.001	0.289	60.329	61.054	60.906
9	197	197	4.893	0.001	0.353	68.765	69.490	69.470
10	192	192	6.211	0.001	0.463	87.906	88.631	88.831
11	190	188	14.969	0.001	0.340	63.319	64.044	64.000
12	183	178	14.209	0.001	0.311	54.736	55.461	55.358
13	168	169	4.506	0.001	0.335	56.004	56.729	56.675
14	167	165	6.576	0.001	0.326	53.105	53.830	53.757
15	166	166	7.178	0.003	0.369	60.478	61.203	61.216
16	137	138	7.021	0.001	0.376	51.134	51.859	51.886
17	136	137	5.583	0.002	0.478	64.559	65.284	65.515
18	137	134	8.914	0.001	0.411	54.272	54.997	55.094
19	130	130	6.799	0.001	0.485	62.051	62.776	63.021
20	128	128	10.776	0.001	0.422	53.204	53.929	54.049
21	125	126	10.013	0.001	0.551	68.287	69.012	69.388
22	124	124	7.900	0.001	0.380	46.314	47.039	47.073
23	124	123	7.471	0.001	0.436	52.733	53.458	53.605
24	121	122	13.093	0.001	0.387	46.385	47.110	47.158
25	104	104	14.271	0.001	0.556	56.689	57.414	57.801
26	96	96	3.828	0.004	0.240	22.565	23.290	23.045
27	85	86	8.327	0.002	0.685	57.517	58.242	58.886
28	84	85	7.183	0.002	0.601	49.865	50.590	51.067
29	82	83	9.39	0.002	0.759	61.439	62.164	62.956
30	40	41	14.250	0.025	1.174	45.786	46.511	48.134
31	29	29	2.886	0.003	0.350	9.445	10.170	10.145
Total	4994	4922	14.969	0.001	0.362	1782.747	1805.222	1808.649

LG, linkage group; Loci refer to genetic ones, not sequence tagged physical RAD loci; Max, maximum; Min, minimum; Avr., Average; Cal., calculated length of a linkage group; Rev. 1, the length of a linkage group revised with the method of Fishman *et al*. [80]; Rev. 2, the length of a linkage group revised with the method of Chakravarti *et al*. [81].

### Characterization of SNP at mapped physical RAD loci

**Figure 4:**
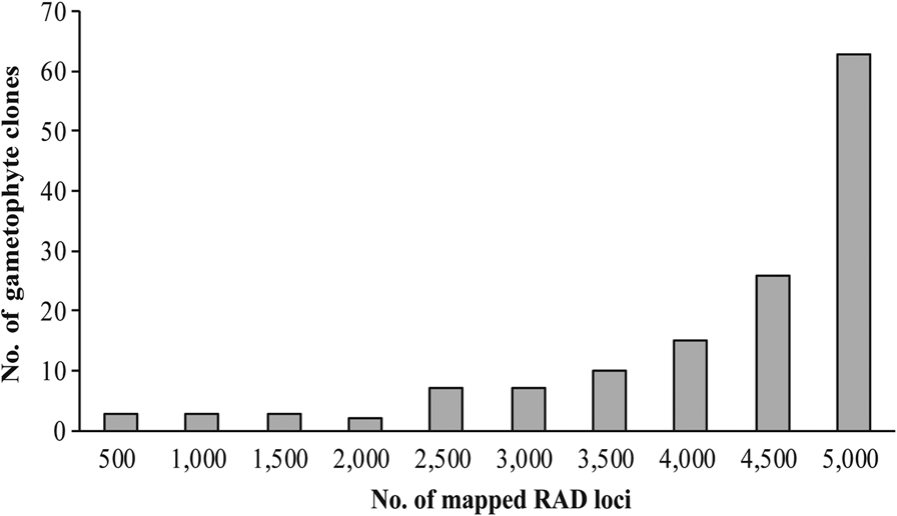
**The number of gametophyte clones with different numbers of mapped RAD loci.**

**Figure 5:**
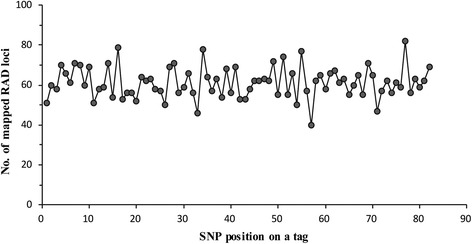
**The number of SNPs found at different nucleotide positions of RAD tags.**

Gametophyte clones contained different numbers of quality physical RAD loci. About 21.6% of quality physical RAD loci were absent in average among these clones. As illustrated in Figure 3, the number of mapped physical RAD loci varied among clones. As showed in Figure 4, only seven clones were genotyped at <3,000 physical RAD loci, and 63 clones were genotyped at >5,000 physical RAD loci. The remaining clones were genotyped at physical RAD loci varying between 3,000 and 5,000. All types of SNP were identified at mapped physical loci. Along 85 nucleotide tags, SNP distributed almost evenly (Figure 5).

### Extension of Taq I associated DNA and microsatellites development

At 4,992 of 4,994 physical RAD loci, *Taq* I associated DNA was extended with Illumina Miseq PE250. The number of trimmed extending reads at these loci ranged from 552 to 4,677 with an average of 897 (Additional file 3). These extended DNA will expand the application of these SNPs to *e.g.* the development of oligonucleotide chips.

The extended tags have allowed us to develop a set of microsatellites for determining genetic diversity, evaluating genetic resources and among others. Microsatellites searching against extended *Taq* I associated DNA at 4,992 physical RAD loci yielded 99 microsatellites (Additional file 4). The simple sequence of these microsatellites was 2–6 bases in length and repeated ≥10 (2n), ≥7 (3n), ≥5 (4n), ≥4 (5n) and ≥3 (6n) times, respectively. These microsatellites were bounded at least 50 nucleotides upstream and downstream, respectively, so that primers can be designed conveniently. Unfortunately, the amplification and polymorphism between two parental gametophyte clones and among conserved kelp gametophyte clones have not been tested. The positions of the *Taq* I associated DNA from them the microsatellites were identified have added locating the microsatellites at the SNP linkage map constructed in this study (Additional files 2 and 4 in red color).

### Mapping of a gametophyte sex determining locus

After calculating SNP linkage map, we mixed the sex trait of kelp gametophyte clones with SNP genotypes of physical RAD loci, grouped these loci and sex trait again and calculated the linkage of gametophyte sex trait and physical RAD loci. It was found that a sex determining locus was mapped on linkage group 2, which was 2.66 cM up to marker_40567 and 6.42 cM down to marker_23595 (Additional files 5 and 6 in red color). Linkage group 2 was the longest, expanding a genetic distance of 92.6 cM. Recalculated linkage group containing sex determining locus was 93.0 cM in length. The sex determining locus located within a window of about 9.0 cM in length.

## Discussion

### SNP linkage map and its application

Kelp has been intensively cultured in China for almost a century. Its genetic improvement is comparable with that of rice in terms of breeding strategy. For example, utilizing heterosis by developing kelp hybrids has increased the unit area yield by 60-70% on the basis of elite varieties [3-5]. In addition, a set of traits, both the biological (*e.g*. gametophyte gender) and the economic (*e.g*. yield and stress tolerance) have been measured frequently during breeding. It is clear that the genetic bases of these traits should be deciphered with molecular tools so that molecular marker assisted breeding could be implemented. Unfortunately, the development of molecular tools for kelp is extremely limited. A few marker systems have been used in kelp [63-69]; however they were either low throughput or not transferable among populations or number limited. Linkage maps of kelp have been tentatively constructed (*e.g*., [64]); unfortunately the markers on these maps are mainly AFLPs which are not transferable among populations.

In this study, we constructed a high density SNP linkage map by sequencing *Taq* I site associated DNA. In total, 4,994 SNPs were assigned to 31 linkage groups. The map expanded a total genetic distance of 1782.75 cM, covering 98.66% of the expected (1806.94 cM). To our knowledge, this is the highest density molecular marker linkage map of kelp constructed to date. The length of RAD tags were 85 bp in length, These tags themselves may serve as interrogators of their homologs; however they cannot serve as the templates of SNP detecting tools, for example, PCR based methods. In order to overcome this shortage, we extended the tag to 400–500 bp with Miseq method. The extended will retain the advantage of tags in homologs searching and offer us easiness in developing SNP detecting tools, and further deciphering the genetic bases of economic traits and cloning their controlling genes. The number of linkage groups we obtained was in accordance with that determined with an improved staining method [21] and DAPI staining [22-24]. Sex specific markers including inter-SSRs [24] and a sequence [22] have been identified. The mapping of kelp gametophyte sex locus and these early findings in combination may aid to approaching the mechanism underlining the sex determination of kelp gametophytes.

SNP genotyping is less efficient with traditional methods, *e.g.* tetra-primer ARMS PCR [27], capillary electrophoresis [28], high resolution melting [29], mass spectrometry [30,31], optical amplification of cationic conjugated polymers and the single base primer extension reaction [32], and PCR and sequencing [40]. Such efficiency may be improved with relatively high throughput methods, *e.g*. multiplex PCR [39], KASPar [41] and GoldenGate [42-45]; however these methods need specifically developed facilities and detergents. Pyrosequencing [37,38], resequencing [46] and even the reduction of genome complexity [50] and RAD sequencing [36,51-53] and ddRAD [54-56,83] are still expensive at this moment. To our knowledge, DNA microarray [33-36] is the most appropriate tool for SNP genotyping as was tried in genomic breeding of rice [61] and characterization of genomic diversity of wheat [62]. We mapped 4,994 RAD physical loci and extended these tags to 400–500 bp in length. This will allow us easiness of developing SNP chips, thus shifting SNP genotyping to a fast, cheap and high throughput track.

Although a long time effort has been made, the kelp microsatellites isolated and mapped were extremely scarce [23,66,67,69]. In this study, we identified a set of microsatellites, 99 in total, from the mapped and extended RAD tags following relatively strict standards, which distributed on all SNP linkage groups (Additional files 2 and 4 in red color). These microsatellites will certainly fill in the gap between traditional molecular markers and high throughput SNP array or SNP chip, thus facilitating a set of works, for example, the evaluation of genetic resources and decipherment of the genetic bases of important traits. The most prominent advantage of this set of microsatellites was the certainty of their position on the linkage map.

### Sex determination of kelp gametophyte

Different sets of genes governing the biosyntheses of ethylene, jasmonic acid, brassinosteroids or gibberellins or occasionally proteins have been identified as the controller of the gender of monoecious plant flowers while a sex determining region (locus) usually evolves to control the gender of dioecious plants [84]. The complexity of sex determining regions varied among a wide range of species between the simplest in yeast where only a gene and its expression regulation elements exist [85] and the most complex, the sex chromosomes in papaya [86-88]. Between the extremes of complexities are sex-determining loci governing the gender of *Chlamydomonas* [89,90], *Volvox* [91,92] and asparagus [93]. The characteristics shared by these loci include the chromosomal rearrangement and avoidance of genetic recombination and the difference in gene content and expression between genders [92]. The life cycles of liverwort and moss are monoploidy gametophytes dominant. In these haploidy systems, sex chromosomes have evolved [94,95]. Pheromone involve in the recognition and fusion of algal gametes, but not the gamete development and sex determination [96]. Fungi belonged to the unikonts, a supergroup of eukaryotes including animals and humans ourselves while green algae belonged to the plantae; and brown algae belonged to chromalveolates [97,98]. The complexity of sex determining loci varies among and within these eukaryotic supergroups.

Eukaryotic analyses have showed that the sex determining locus containing chromosomes (sex chromosomes) of kelp are morphologically identical [21-24]. In this study, a sex determining locus was mapped on linkage group 2 in a window about 9.0 cM in width (2.66 cM up to marker_40567 and 6.42 cM down to marker_23595). Mapping of the sex determining locus of kelp gametophytes stepped one pace at least toward the structure of this locus and its comparison with those of other species. Recently, the structure of the sex determination locus of model brown alga *Ectocarpus* has been described [99]. Fine mapping this locus and describing its structure thus understanding the sex determination of kelp gametophytes will be the focus of our future studies. Although a long way ahead, we will model our future works on what have achieved in *Ectocarpus*.

## Conclusions

A high density SNP linkage map was constructed for kelp, an intensively cultured brown alga in China. On 31 linkage groups, 4,994 SNP-containing tag defined RAD loci were mapped. The map expanded a total distance of 1,782.75 cM, covering 98.66% of the expected. The number of linkage groups was in accordance with that of real chromosomes. The length of RAD tags was extended to about 400–500 bp so that SNP based tools, *e.g.* SNP chips, can be developed. In addition, 99 microsatellites were identified among extended RAD tags. A sex determining locus was mapped on linkage group 2. This map will facilitate the studies on kelp genes, genetics and genomics, and may provide a reference for those studies in *Volvox*, mosses and ferns.

### Availability of supporting data

The RAD reads of 139 gametophytes as a mapping panel and 2 parental gametophytes of kelp (*Saccharina japonica*) have been submitted to NCBI under a bioproject accession number PRJNA274218 (http://www.ncbi.nlm.nih.gov/bioproject/274218) which links to 141 Sequence Read Archive (SRA) accessions corresponding to 141 gametophytes, respectively. The mapped RAD tags with SNP marked have also been listed in Additional file 1. The extensions of mapped RAD tags have been listed in Additional file 3.

## Additional files

Additional file 1:
**Tabulated SNP linkage map constructed in this study.** The name of markers, the sequence of RAD tags, and the recombination value between markers are listed. Additional file 2:
**Graphical SNP linkage map constructed in this study with the loci containing microsatellites marked red.** The mapped SNP markers, their position on linkage groups and genetic distance in cM are illustrated. Additional file 3:
**Tag sequences extended with Miseq.** In order to amplify the applicability of the map constructed to, for example, the development of SNP genotyping tools other than sequencing, the mapped RAD tags are extended. The listed include the extended tag sequences and their corresponding marker names. Additional file 4:
**Microsatellites identified in extended tag sequences and their characteristics.** In total, 99 microsatellites were identified among the extended tag sequences. These microsatellites are applicable to diverse studies as their location on the map is known. Additional file 5:
**Tabulated position of the sex determining locus of kelp gametophytes.** In this table, the sex determining locus and its position on linkage group 2 are listed. Additional file 6:
**Graphical position of the sex determining locus of kelp gametophytes.** The illustrated include the sex determining locus (in red color) and its relationship to the adjacent markers on linkage group 2.

## Abbreviations

SNP: Single nucleotide polymorphism
RAD: Restriction site associated DNA
DAPI: 4,6-diamino-2-phenyl indole
SSR: Simple sequence repeat
T-ARMS-PCR: Tetra primer amplification refractory mutation system - polymerase chain reaction
KASPar: Competitive allele specific PCR
ddRAD: Double digest RAD
AFLP: Amplified fragment length polymorphism
QTL: Quantitative trait locus
CTAB: Cetyltriethylammnonium bromide
EDTA: Ethylene diamine tetraacetic acid
LOD: Log of odds
BAC: Bacterial artificial chromosome
