# Supplementary material for: Construction of a high density SNP linkage map of kelp (Saccharina japonica) by sequencing Taq I site associated DNA and mapping of a sex determining locus
Source: BMC Genomics. 2015 Mar 15;16(1):189. doi: 10.1186/s12864-015-1371-1 (PMC4369078; doi:10.1186/s12864-015-1371-1)
Supplement: Additional file 2: — Graphical SNP linkage map constructed in this study with the loci containing microsatellites marked red. The mapped SNP markers, their position on linkage groups and genetic distance in cM are illustrated. [file 12864_2015_1371_MOESM2_ESM.pdf]

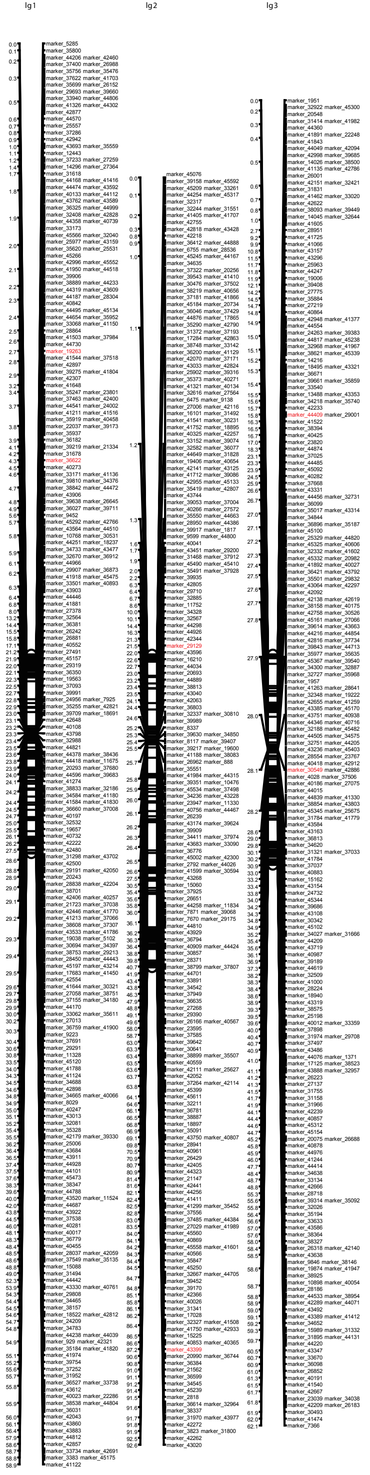
lg1
lg2
lg3

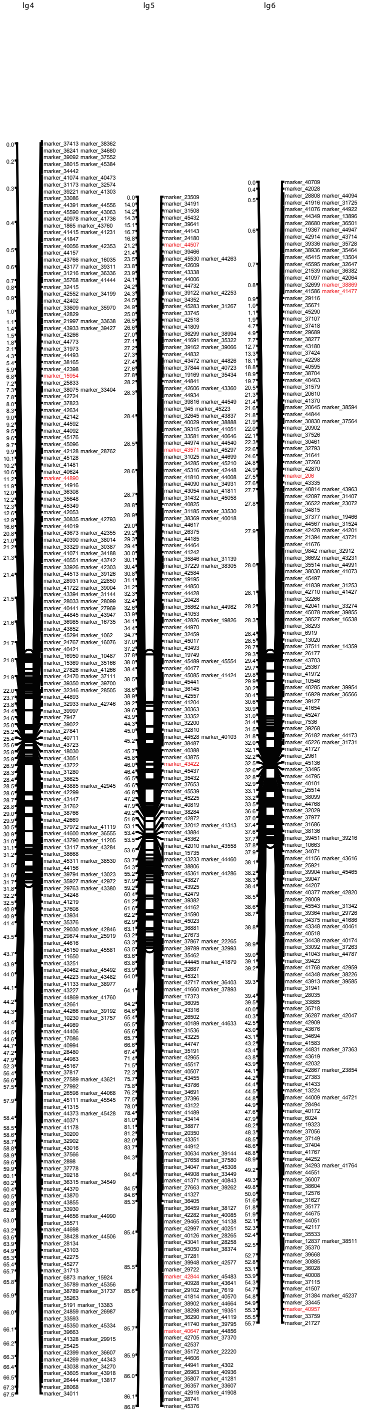
lg4
lg5
lg6

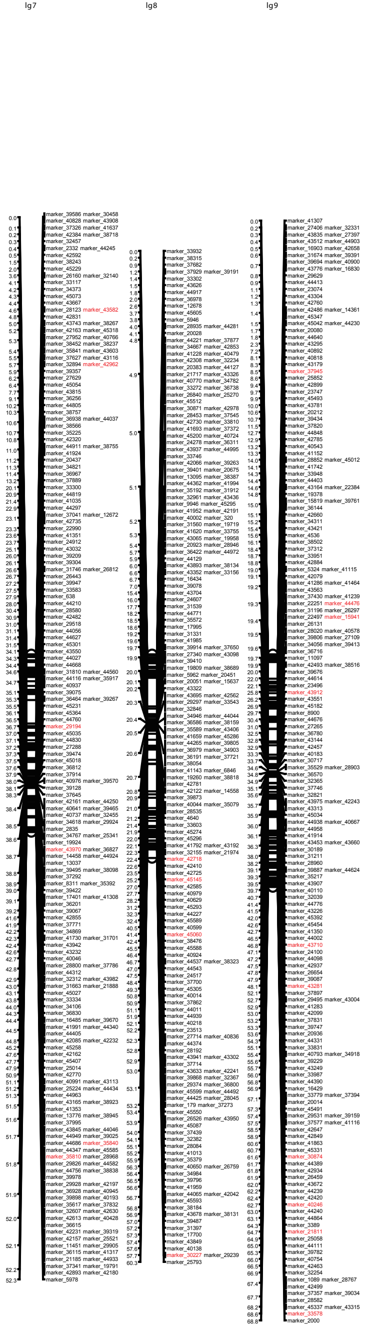
lg7
lg8
lg9

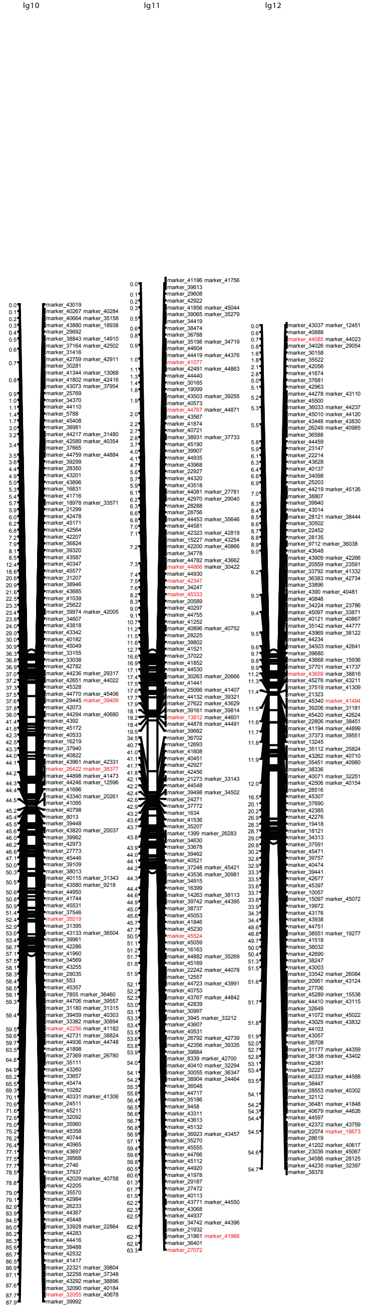
lg10
0.0 0.1 0.2 0.3 0.4 0.5 0.6 0.7 0.8 0.9 1.0 1.1 1.4 1.7 3.0 3.2 3.4 3.5 3.6 4.4 4.9 5.0 5.3 5.4 5.7 5.9 6.0 6.2 6.8 7.2 7.9 8.1 8.5 12.4 18.6 20.6 20.9 21.6 22.5 23.3 23.4 23.8 24.0 29.0 30.0 30.9 36.3 36.8 36.9 37.0 37.2 37.3 37.5 37.6 37.8 38.0 41.4 41.8 42.3 42.5 43.3 44.0 44.1 44.2 44.3 44.4 44.5 45.2 45.4 45.5 45.6 46.0 46.2 46.6 47.1 50.0 50.3 50.5 50.6 50.8 50.9 51.4 52.4 52.9 53.0 53.4 56.7 57.1 57.5 58.1 58.3 58.4 58.5 59.1 59.3 59.4 59.5 59.6 59.7 63.5 64.8 64.9 65.2 65.5 69.3 70.1 70.6 71.6 72.6 75.0 75.2 76.2 76.4 77.1 77.5 77.7 78.5 78.8 79.0 79.1 82.9 83.9 84.5 85.0 85.2 85.3 85.6 85.7 86.5 86.9 87.1 87.6 87.7 87.9
marker_43019
marker_40267 marker_40284
marker_40664 marker_35158
marker_43880 marker_18938
marker_29692
marker_38843 marker_14910
marker_37164 marker_42502
marker_31416
marker_42759 marker_42911
marker_30281
marker_41344 marker_13068
marker_41802 marker_42416
marker_43073 marker_37954
marker_25769
marker_34370
marker_44110
marker_5788
marker_45408
marker_38981
marker_44217 marker_31480
marker_42589 marker_40354
marker_37665
marker_44759 marker_44884
marker_39299
marker_28350
marker_43201
marker_43896
marker_16831
marker_41716
marker_18978 marker_33571
marker_21299
marker_42478
marker_45171
marker_42564
marker_42207
marker_36824
marker_39320
marker_43587
marker_40347
marker_45577
marker_31207
marker_38946
marker_43685
marker_41039
marker_25622
marker_39974 marker_42005
marker_34607
marker_43818
marker_43342
marker_40182
marker_45049
marker_33155
marker_33038
marker_42782
marker_44236 marker_29317
marker_42651 marker_44022
marker_45328
marker_44770 marker_45406
marker_29445 marker_39409
marker_42073
marker_44294 marker_40680
marker_4392
marker_45172
marker_40533
marker_16219
marker_37940
marker_40822
marker_43961 marker_42331
marker_26422 marker_38377
marker_44898 marker_41473
marker_44246 marker_12596
marker_41696
marker_43340 marker_20261
marker_41095
marker_40798
marker_8013
marker_39448
marker_43820 marker_20037
marker_39962
marker_42973
marker_27773
marker_45446
marker_39109
marker_38013
marker_40115 marker_31343
marker_43580 marker_9218
marker_44950
marker_41744
marker_45531
marker_37546
marker_35519
marker_31395
marker_43133 marker_36504
marker_39961
marker_42286
marker_41960
marker_34569
marker_43255
marker_29035
marker_553
marker_45357
marker_7855 marker_36460
marker_44706 marker_39557
marker_31180 marker_31315
marker_39459 marker_40303
marker_33382 marker_30894
marker_42256 marker_41182
marker_42731 marker_38824
marker_44936 marker_44748
marker_41898
marker_27369 marker_26780
marker_35111
marker_43260
marker_33657
marker_45474
marker_10282
marker_40331 marker_41306
marker_24511
marker_45211
marker_32092
marker_35960
marker_45358
marker_40744
marker_40965
marker_43697
marker_39568
marker_2746
marker_37937
marker_42029 marker_40758
marker_42205
marker_35570
marker_42984
marker_26233
marker_44367
marker_45448
marker_33928 marker_22864
marker_44283
marker_44416
marker_39488
marker_42532
marker_41417
marker_22321 marker_39804
marker_32258 marker_37348
marker_43292 marker_38896
marker_32090 marker_40184
marker_32055 marker_40678
marker_39992
lg11
0.0 0.1 0.2 0.3 0.5 0.6 0.8 0.9 1.0 1.1 1.3 1.4 1.8 1.9 2.0 2.2 2.7 2.8 3.7 4.0 4.1 4.7 5.6 5.7 5.9 6.1 6.2 6.3 6.6 6.8 7.0 7.1 7.2 7.3 7.4 7.5 7.6 8.2 8.3 9.0 9.1 10.7 11.2 11.5 11.6 12.7 16.6 16.9 17.1 17.4 17.5 17.6 17.7 17.9 18.1 18.2 18.4 19.5 34.5 40.7 41.0 41.1 41.7 41.8 42.1 42.2 42.4 42.6 42.9 43.2 43.6 43.7 43.8 43.9 44.0 44.1 44.2 44.3 44.4 44.6 44.9 45.0 45.6 45.7 47.7 50.5 51.1 51.2 51.7 51.8 51.9 52.1 52.2 52.3 53.0 53.1 53.3 53.4 53.5 53.6 53.8 53.9 54.0 54.1 54.2 55.3 55.8 56.4 56.5 56.6 56.7 56.8 56.9 57.1 59.4 59.6 59.8 60.5 60.6 61.3 61.7 61.9 62.2 62.3 62.5 62.6 62.7 62.9 63.3
marker_41196 marker_41756
marker_39613
marker_29608
marker_42922
marker_41856 marker_45044
marker_39065 marker_35279
marker_34419
marker_38474
marker_36788
marker_35198 marker_34719
marker_44604
marker_44419 marker_44376
marker_41077
marker_42491 marker_44863
marker_44440
marker_30165
marker_19099
marker_43503 marker_39255
marker_40573
marker_44767 marker_44871
marker_43567
marker_41874
marker_40721
marker_38931 marker_37733
marker_45190
marker_39907
marker_44935
marker_43968
marker_22927
marker_44320
marker_43518
marker_44081 marker_27781
marker_42970 marker_29040
marker_28288
marker_28756
marker_44453 marker_35646
marker_44581
marker_42323 marker_42819
marker_15227 marker_43254
marker_42200 marker_40866
marker_34778
marker_44782 marker_43662
marker_44866 marker_30422
marker_44930
marker_42347
marker_34247
marker_45333
marker_20589
marker_40297
marker_44755
marker_41252
marker_40896 marker_40752
marker_28225
marker_39802
marker_41521
marker_37022
marker_41852
marker_44530
marker_30263 marker_20666
marker_41441
marker_25066 marker_41407
marker_44132 marker_39321
marker_27622 marker_43629
marker_39161 marker_39814
marker_13812 marker_44601
marker_44878 marker_44491
marker_39662
marker_36702
marker_12693
marker_41608
marker_40451
marker_42927
marker_42456
marker_21273 marker_33143
marker_44548
marker_39498 marker_34502
marker_24271
marker_37772
marker_1634
marker_41536
marker_35207
marker_1399 marker_26283
marker_34630
marker_33678
marker_39462
marker_40521
marker_37248 marker_45421
marker_43536 marker_30981
marker_34915
marker_16399
marker_14263 marker_38113
marker_39742 marker_44395
marker_38737
marker_45053
marker_41846
marker_45230
marker_45524
marker_45059
marker_16163
marker_44882 marker_35269
marker_45169
marker_22242 marker_44078
marker_12557
marker_44723 marker_43991
marker_40753
marker_43767 marker_44842
marker_42839
marker_30997
marker_3945 marker_33212
marker_43607
marker_40531
marker_26792 marker_42739
marker_42356 marker_39335
marker_39884
marker_8339 marker_42700
marker_40410 marker_32294
marker_30055 marker_36347
marker_38904 marker_24464
marker_36548
marker_44717
marker_35186
marker_9458
marker_43311
marker_43613
marker_45132
marker_36923 marker_43457
marker_35270
marker_45555
marker_44766
marker_45112
marker_44920
marker_41978
marker_29187
marker_27472
marker_40113
marker_43771 marker_44550
marker_43068
marker_44937
marker_34742 marker_44396
marker_21932
marker_31861 marker_41966
marker_36401
marker_27072
lg12
0.0 0.6 0.7 0.8 1.6 1.8 2.1 2.8 5.0 5.1 5.2 5.3 5.5 5.8 5.9 6.1 6.3 6.4 6.6 6.9 7.0 8.3 8.4 8.5 8.6 8.7 8.8 8.9 9.0 9.2 9.3 9.4 9.5 9.6 9.7 9.8 9.9 11.2 11.3 11.4 11.5 11.6 11.7 11.8 11.9 12.0 16.5 20.1 20.2 26.9 28.3 28.7 29.0 29.8 30.2 32.8 32.9 33.3 33.5 33.6 33.7 33.8 33.9 34.3 34.4 48.6 48.8 49.7 50.0 50.4 51.3 51.5 51.6 51.7 51.8 51.9 52.0 52.7 52.8 53.1 53.4 53.5 54.1 54.2 54.3 54.4 54.5 54.6 54.7
marker_43037 marker_12451
marker_40888
marker_44085 marker_44023
marker_34026 marker_29054
marker_30158
marker_35522
marker_42056
marker_41674
marker_37681
marker_42963
marker_44278 marker_43110
marker_45500
marker_36033 marker_44237
marker_45010 marker_44120
marker_43448 marker_43830
marker_26249 marker_40985
marker_36588
marker_44459
marker_23147
marker_43628
marker_22214
marker_40137
marker_34098
marker_25203
marker_44219 marker_45126
marker_38807
marker_39940
marker_43014
marker_28121 marker_38444
marker_30502
marker_22452
marker_28135
marker_9712 marker_36038
marker_43648
marker_43909 marker_42266
marker_20559 marker_23591
marker_33792 marker_41332
marker_36383 marker_42734
marker_33896
marker_4390 marker_40481
marker_40848
marker_34224 marker_23786
marker_45097 marker_33871
marker_40121 marker_40867
marker_35142 marker_44777
marker_43969 marker_38122
marker_44234
marker_34503 marker_42641
marker_39680
marker_43668 marker_15936
marker_37701 marker_41737
marker_43659 marker_38816
marker_45276 marker_43211
marker_37519 marker_41309
marker_21323
marker_45340 marker_41494
marker_39206 marker_31181
marker_45420 marker_42624
marker_22806 marker_38451
marker_41194 marker_44899
marker_37373 marker_39551
marker_13245
marker_35112 marker_25824
marker_43262 marker_40710
marker_35451 marker_40980
marker_38336
marker_40071 marker_32251
marker_42506 marker_40154
marker_28516
marker_45307
marker_37690
marker_42385
marker_42276
marker_19418
marker_18121
marker_34313
marker_37591
marker_45471
marker_39757
marker_40474
marker_39441
marker_42677
marker_45397
marker_10057
marker_15097 marker_45072
marker_19972
marker_43176
marker_43938
marker_44751
marker_38551 marker_19277
marker_41518
marker_38032
marker_42690
marker_38247
marker_43003
marker_33542 marker_26084
marker_20951 marker_43124
marker_27706
marker_45289 marker_15538
marker_44410 marker_43115
marker_32649
marker_41072 marker_45022
marker_43029 marker_43832
marker_44103
marker_43057
marker_38708
marker_31177 marker_44359
marker_38138 marker_43402
marker_42381
marker_32227
marker_40333 marker_44588
marker_38447
marker_28553 marker_40302
marker_32112
marker_36481 marker_41848
marker_40679 marker_44626
marker_44597
marker_42372 marker_43759
marker_22074 marker_18673
marker_28619
marker_41202 marker_40617
marker_23036 marker_45067
marker_34586 marker_28125
marker_44235 marker_32397
marker_38378

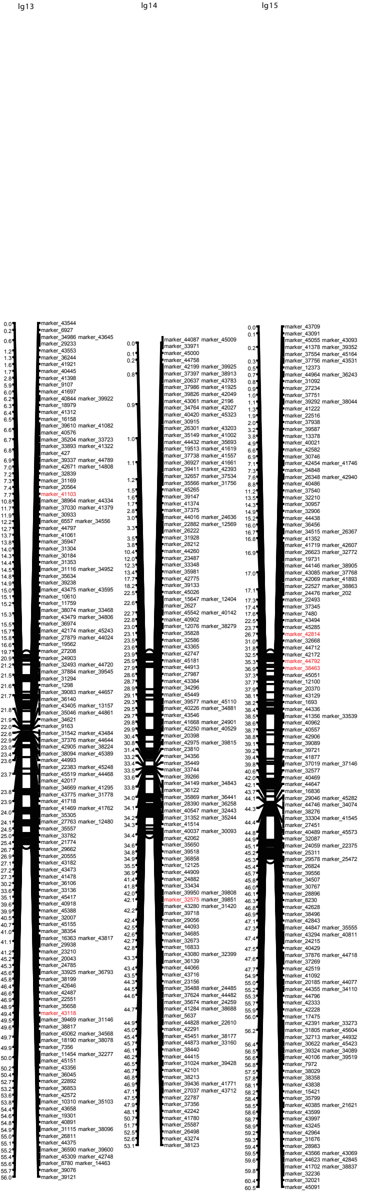
lg13
lg14
lg15
0.0
0.2
0.6
1.2
1.3
1.6
1.7
2.8
5.9
6.0
6.2
6.3
6.4
6.5
6.6
6.7
6.8
6.9
7.0
7.2
7.3
7.4
7.7
10.8
11.7
11.9
12.2
12.7
13.7
13.8
14.0
14.3
14.4
14.5
14.8
14.9
15.0
15.1
15.2
15.3
15.5
15.7
15.8
16.6
19.7
20.9
21.2
21.5
21.6
21.7
21.8
21.9
22.0
22.6
22.9
23.0
23.4
23.6
23.7
23.8
23.9
24.0
24.1
24.5
25.0
25.4
26.7
26.9
27.1
27.2
27.3
30.2
33.6
35.1
39.6
39.9
40.5
40.7
41.0
41.1
41.2
45.2
45.3
45.6
45.8
46.4
46.6
46.8
48.9
49.4
49.5
49.6
49.7
49.9
50.0
50.2
50.4
52.7
53.2
53.6
53.8
54.4
54.6
54.8
54.9
55.0
55.1
55.2
55.4
55.6
55.7
56.0
marker_43544
marker_6927
marker_34986 marker_43645
marker_29233
marker_43553
marker_36244
marker_41921
marker_40445
marker_41398
marker_9107
marker_41697
marker_40844 marker_39922
marker_18979
marker_41312
marker_16158
marker_39610 marker_41082
marker_40576
marker_35204 marker_33723
marker_33893 marker_41322
marker_427
marker_39337 marker_44789
marker_42671 marker_14808
marker_32839
marker_31169
marker_20564
marker_41103
marker_38964 marker_44334
marker_37030 marker_41379
marker_30933
marker_6557 marker_34556
marker_44797
marker_41061
marker_35947
marker_31304
marker_30184
marker_31353
marker_31116 marker_34952
marker_35634
marker_39238
marker_43475 marker_43595
marker_10610
marker_11759
marker_38074 marker_33468
marker_43479 marker_34806
marker_36974
marker_42174 marker_45243
marker_27879 marker_44024
marker_19562
marker_27208
marker_24903
marker_32493 marker_44720
marker_37884 marker_39545
marker_31294
marker_1298
marker_39083 marker_44657
marker_36140
marker_43405 marker_13157
marker_35046 marker_44861
marker_34621
marker_9163
marker_31542 marker_43484
marker_37376 marker_44644
marker_42905 marker_38224
marker_38094 marker_45389
marker_44993
marker_22383 marker_45248
marker_45519 marker_44468
marker_42017
marker_34669 marker_41295
marker_43775 marker_31778
marker_41718
marker_41469 marker_41762
marker_35305
marker_27763 marker_12480
marker_35557
marker_33782
marker_21774
marker_29662
marker_20555
marker_43182
marker_43473
marker_41478
marker_36106
marker_33136
marker_45417
marker_40918
marker_45388
marker_32007
marker_45155
marker_38354
marker_16363 marker_43817
marker_29938
marker_23210
marker_20043
marker_24785
marker_33925 marker_36793
marker_38199
marker_42646
marker_42487
marker_22551
marker_35658
marker_43118
marker_39469 marker_31146
marker_38817
marker_45062 marker_34568
marker_18190 marker_38078
marker_7356
marker_11454 marker_32277
marker_45151
marker_43356
marker_36045
marker_22892
marker_36853
marker_42572
marker_10310 marker_35103
marker_43658
marker_19301
marker_40891
marker_31115 marker_38096
marker_26811
marker_44375
marker_36590 marker_39600
marker_45309 marker_42748
marker_8780 marker_14463
marker_39076
marker_39121
0.0
0.1
0.2
0.8
0.9
1.0
1.1
1.2
1.5
1.6
1.7
2.8
3.0
3.3
3.5
3.8
10.4
12.0
12.3
13.4
17.7
18.2
22.5
22.6
22.7
22.8
23.0
23.1
23.5
23.6
23.9
25.9
27.9
28.6
28.7
28.9
29.1
29.3
29.5
29.7
29.8
29.9
30.4
30.8
31.4
33.2
33.4
33.5
33.6
33.8
34.0
34.1
34.2
34.3
34.4
34.6
34.8
34.9
35.5
36.9
41.4
41.8
42.0
42.1
42.2
42.4
42.5
42.6
42.7
42.8
43.3
43.4
43.5
44.3
44.5
44.6
44.7
44.9
45.0
45.4
45.7
46.1
46.2
46.5
46.7
46.9
46.8
47.1
47.5
47.9
48.1
50.7
51.7
52.5
52.6
53.1
marker_44087 marker_45009
marker_33971
marker_45000
marker_44758
marker_42199 marker_39925
marker_37397 marker_38913
marker_20637 marker_43783
marker_37986 marker_41925
marker_39826 marker_42049
marker_43061 marker_2196
marker_34764 marker_42027
marker_40420 marker_45323
marker_30915
marker_26301 marker_43203
marker_35149 marker_41002
marker_44432 marker_35693
marker_19513 marker_41619
marker_37738 marker_41557
marker_36927 marker_41661
marker_39411 marker_42393
marker_32657 marker_37534
marker_35566 marker_31756
marker_45265
marker_39147
marker_41374
marker_37375
marker_44016 marker_24636
marker_22882 marker_12569
marker_26222
marker_31928
marker_28212
marker_44260
marker_23487
marker_33348
marker_35981
marker_42775
marker_39133
marker_45026
marker_15647 marker_12404
marker_2627
marker_45542 marker_40142
marker_40902
marker_12076 marker_38279
marker_35828
marker_32586
marker_43365
marker_42747
marker_45181
marker_44913
marker_27987
marker_43384
marker_34296
marker_45449
marker_39577 marker_45110
marker_40226 marker_34881
marker_43546
marker_41668 marker_24901
marker_42250 marker_40529
marker_20398
marker_42975 marker_39815
marker_23810
marker_34356
marker_35449
marker_33744
marker_39266
marker_34149 marker_34843
marker_36122
marker_35869 marker_36441
marker_28390 marker_36258
marker_40547 marker_32443
marker_31352 marker_35244
marker_41514
marker_40037 marker_30093
marker_42062
marker_35650
marker_39518
marker_36858
marker_12125
marker_44909
marker_24882
marker_33434
marker_39950 marker_39808
marker_32575 marker_39851
marker_43280 marker_31420
marker_39718
marker_29056
marker_44093
marker_34685
marker_32673
marker_16833
marker_43080 marker_32399
marker_36139
marker_44066
marker_43716
marker_23156
marker_35488 marker_24485
marker_37624 marker_44482
marker_35674 marker_24259
marker_41284 marker_38688
marker_5637
marker_44828 marker_22610
marker_42291
marker_45451 marker_38177
marker_44873 marker_33160
marker_38440
marker_44415
marker_31024 marker_39428
marker_42101
marker_38213
marker_39436 marker_41771
marker_27037 marker_43712
marker_22787
marker_37356
marker_42242
marker_41780
marker_25587
marker_26498
marker_43274
marker_38123
0.0
0.1
0.2
0.3
0.5
0.7
0.8
0.9
1.0
1.1
1.3
1.9
2.0
3.2
3.8
4.9
6.2
6.9
7.1
7.3
7.6
8.8
11.2
13.5
14.3
14.9
15.3
16.0
16.7
16.8
16.9
17.0
17.1
17.3
17.4
17.6
22.5
23.7
26.7
31.0
31.8
32.5
35.3
36.9
37.3
37.7
37.9
38.1
38.2
38.4
38.5
38.6
38.7
38.8
39.1
39.2
39.4
39.8
40.6
42.0
42.1
43.7
44.1
44.3
44.4
44.8
44.9
45.1
45.2
45.3
45.4
45.7
45.8
46.0
46.1
46.3
46.8
46.9
47.1
47.3
47.4
47.5
47.6
47.7
54.9
55.0
55.2
55.3
55.7
56.0
56.2
56.4
56.5
56.6
56.8
57.5
57.8
58.1
58.2
58.4
58.5
58.6
58.7
58.9
59.2
59.3
59.4
59.5
59.6
59.8
60.4
60.5
marker_43709
marker_43091
marker_45055 marker_43093
marker_41378 marker_39352
marker_37554 marker_45164
marker_37756 marker_43531
marker_12373
marker_44964 marker_36243
marker_31092
marker_27234
marker_37751
marker_39292 marker_38044
marker_41222
marker_22516
marker_37938
marker_39587
marker_13378
marker_40021
marker_42582
marker_30746
marker_42454 marker_41746
marker_34848
marker_26348 marker_42940
marker_40486
marker_37540
marker_32210
marker_30957
marker_32906
marker_44438
marker_36456
marker_34515 marker_26367
marker_41352
marker_41719 marker_42607
marker_26623 marker_32772
marker_19731
marker_44146 marker_38905
marker_43085 marker_37768
marker_42069 marker_41893
marker_22527 marker_38863
marker_24476 marker_202
marker_22493
marker_37345
marker_7480
marker_43494
marker_45285
marker_42814
marker_32668
marker_44712
marker_42172
marker_44792
marker_38463
marker_45051
marker_12100
marker_20370
marker_43129
marker_1693
marker_44336
marker_41356 marker_33539
marker_40962
marker_40557
marker_42906
marker_39089
marker_39721
marker_41877
marker_37019 marker_37146
marker_32577
marker_40469
marker_44647
marker_16836
marker_29046 marker_45282
marker_44746 marker_34074
marker_38276
marker_33304 marker_41545
marker_27451
marker_40489 marker_45573
marker_32087
marker_24059 marker_22375
marker_25311
marker_29578 marker_25472
marker_26824
marker_39556
marker_34507
marker_30767
marker_28896
marker_8230
marker_42628
marker_38496
marker_42843
marker_44847 marker_35555
marker_43294 marker_40811
marker_24215
marker_40429
marker_37876 marker_44718
marker_37269
marker_42519
marker_41092
marker_20185 marker_44077
marker_44355 marker_34110
marker_44796
marker_42333
marker_42228
marker_17475
marker_42391 marker_33273
marker_31805 marker_45604
marker_32713 marker_44932
marker_30622 marker_45423
marker_39324 marker_34089
marker_40106 marker_39519
marker_7972
marker_38029
marker_38358
marker_43838
marker_15421
marker_35799
marker_40385 marker_21621
marker_43599
marker_43997
marker_43245
marker_42964
marker_31676
marker_28983
marker_43566 marker_43069
marker_44623 marker_42845
marker_41702 marker_38837
marker_32236
marker_32021
marker_45091

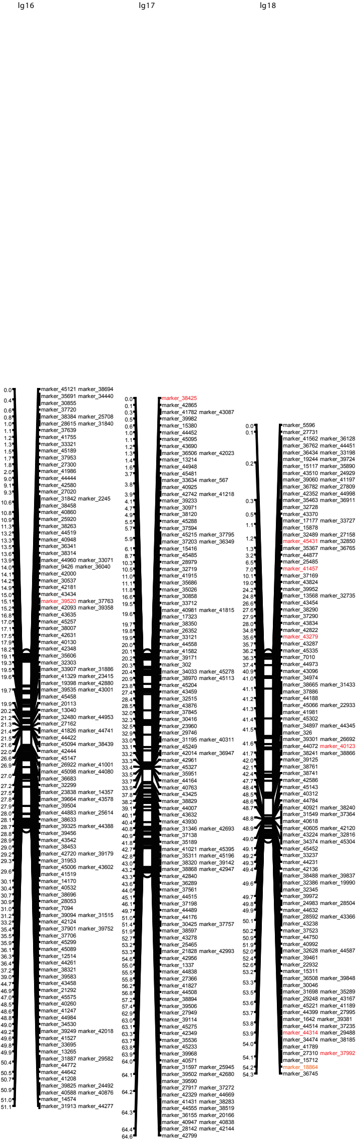
lg16
lg17
lg18
0.0
0.4
0.6
0.8
1.0
1.1
1.2
1.3
1.5
1.7
1.8
2.0
9.0
9.1
9.3
10.6
10.8
10.9
11.3
13.2
13.3
13.5
13.6
13.9
14.0
14.1
14.2
14.9
15.0
15.1
15.2
16.8
17.0
17.1
17.5
17.9
18.2
19.1
19.3
19.5
19.6
19.7
19.9
20.2
21.2
21.3
21.4
21.5
21.6
22.0
26.6
26.9
27.0
27.2
27.5
27.8
27.9
28.0
28.1
28.7
28.8
28.9
29.0
29.2
29.3
29.6
30.1
30.4
30.7
30.9
31.0
31.2
33.2
35.4
35.5
35.9
36.1
36.3
36.4
37.2
39.0
44.7
46.9
47.0
48.5
48.6
48.8
49.0
49.2
49.6
49.8
49.9
50.4
50.5
50.7
50.9
51.0
51.1
marker_45121 marker_38694
marker_35691 marker_34440
marker_30855
marker_37720
marker_38384 marker_25708
marker_28615 marker_31840
marker_37639
marker_41755
marker_33321
marker_45189
marker_37953
marker_27300
marker_41986
marker_44444
marker_42580
marker_27020
marker_31842 marker_2245
marker_38458
marker_40860
marker_25920
marker_38263
marker_44519
marker_40948
marker_36341
marker_38314
marker_44960 marker_33071
marker_9426 marker_36040
marker_42000
marker_30537
marker_42181
marker_43434
marker_39520 marker_37763
marker_42093 marker_39358
marker_43635
marker_45257
marker_38007
marker_42631
marker_40130
marker_42348
marker_35606
marker_32303
marker_33907 marker_31886
marker_41329 marker_23415
marker_19398 marker_42880
marker_39535 marker_43001
marker_45458
marker_20113
marker_13040
marker_32480 marker_44953
marker_27162
marker_41826 marker_44741
marker_44422
marker_45094 marker_38439
marker_42444
marker_45147
marker_26922 marker_41001
marker_45098 marker_44080
marker_36683
marker_32299
marker_23838 marker_14357
marker_39664 marker_43578
marker_39504
marker_44883 marker_25614
marker_38633
marker_39325 marker_44388
marker_39456
marker_43542
marker_38453
marker_42720 marker_39179
marker_31953
marker_45006 marker_43602
marker_41519
marker_14170
marker_40532
marker_38696
marker_28053
marker_7094
marker_39094 marker_31515
marker_42124
marker_37901 marker_39752
marker_37706
marker_45299
marker_45089
marker_12514
marker_44261
marker_38321
marker_39583
marker_43458
marker_21292
marker_45575
marker_40260
marker_41247
marker_44984
marker_34530
marker_39249 marker_42018
marker_41527
marker_33695
marker_13265
marker_31887 marker_29582
marker_44772
marker_44642
marker_41208
marker_39825 marker_24492
marker_40588 marker_40876
marker_14574
marker_31913 marker_44277
0.0
0.1
0.3
0.5
0.6
1.0
1.1
1.2
1.3
1.4
1.6
3.7
3.8
3.9
4.1
4.7
4.9
5.5
5.7
5.9
6.1
8.7
10.3
10.5
11.0
11.1
11.8
16.6
19.5
19.6
19.7
19.8
19.9
20.0
20.1
20.2
20.3
20.4
20.9
23.8
27.4
28.4
28.5
32.0
32.3
32.5
32.9
33.0
33.1
33.2
33.3
33.4
33.5
33.7
33.9
37.8
38.2
39.1
40.1
40.4
40.8
40.9
41.8
42.7
42.8
43.0
43.2
43.3
43.6
44.0
45.1
46.1
49.7
51.0
51.4
51.9
52.7
53.2
53.3
54.6
55.0
55.5
55.8
56.2
56.8
57.2
57.4
62.9
63.0
63.1
63.3
63.7
63.8
63.9
64.0
64.1
64.2
64.3
64.4
64.6
marker_38425
marker_42865
marker_41782 marker_43087
marker_39982
marker_15380
marker_44452
marker_45095
marker_43690
marker_36506 marker_42023
marker_13214
marker_44948
marker_45481
marker_33634 marker_567
marker_40925
marker_42742 marker_41218
marker_39233
marker_30971
marker_38120
marker_45288
marker_37594
marker_45215 marker_37795
marker_37203 marker_36349
marker_15416
marker_45485
marker_28979
marker_32719
marker_41915
marker_35686
marker_35026
marker_30858
marker_33712
marker_40981 marker_41815
marker_17323
marker_38350
marker_26352
marker_33121
marker_44558
marker_41582
marker_39171
marker_302
marker_34033 marker_45278
marker_38970 marker_45113
marker_45204
marker_43459
marker_32515
marker_43876
marker_37845
marker_30416
marker_23960
marker_29746
marker_31195 marker_40311
marker_45249
marker_42014 marker_36947
marker_42961
marker_45327
marker_35951
marker_44164
marker_40763
marker_43425
marker_38829
marker_44007
marker_43632
marker_43930
marker_31346 marker_42693
marker_37138
marker_35189
marker_41021 marker_45395
marker_35311 marker_45196
marker_38320 marker_39142
marker_38868 marker_42947
marker_42840
marker_36289
marker_37561
marker_44515
marker_37198
marker_44489
marker_44176
marker_30425 marker_37757
marker_38597
marker_43278
marker_25465
marker_21828 marker_42993
marker_42956
marker_1337
marker_44838
marker_27366
marker_41827
marker_44508
marker_38894
marker_39506
marker_27949
marker_39114
marker_45275
marker_42349
marker_35536
marker_45233
marker_39968
marker_40571
marker_31597 marker_25945
marker_39502 marker_42680
marker_39590
marker_27917 marker_37272
marker_42329 marker_44669
marker_41431 marker_38283
marker_44555 marker_38519
marker_36155 marker_20166
marker_40947 marker_40838
marker_28142 marker_42144
marker_42799
0.0
0.1
0.2
0.3
0.5
1.1
1.2
1.3
3.2
6.5
7.0
10.1
19.0
24.2
24.8
26.6
27.6
27.9
28.0
34.8
35.6
35.7
36.2
36.3
37.4
40.9
41.0
41.1
41.2
41.3
41.4
41.5
41.6
41.7
42.0
42.1
42.4
47.7
48.4
48.5
48.6
48.7
48.8
48.9
49.0
49.1
49.2
49.3
49.4
49.6
49.7
49.8
49.9
50.1
50.2
50.9
51.9
52.0
52.4
52.6
53.2
53.3
53.5
53.6
53.7
53.8
53.9
54.0
54.1
54.2
54.3
marker_5596
marker_27731
marker_41562 marker_36128
marker_36762 marker_44451
marker_36434 marker_33198
marker_19244 marker_39724
marker_15117 marker_35890
marker_43510 marker_24929
marker_39060 marker_41197
marker_36782 marker_27809
marker_42352 marker_44998
marker_35463 marker_36911
marker_32728
marker_43370
marker_17177 marker_33727
marker_15878
marker_32489 marker_27158
marker_45431 marker_32850
marker_35367 marker_36765
marker_44877
marker_25485
marker_41457
marker_37169
marker_43824
marker_39952
marker_13568 marker_32735
marker_43454
marker_38290
marker_37290
marker_43834
marker_42822
marker_43279
marker_43287
marker_45335
marker_7010
marker_44973
marker_43096
marker_34974
marker_38665 marker_31433
marker_37886
marker_44188
marker_45066 marker_22933
marker_41981
marker_45302
marker_34897 marker_44345
marker_326
marker_39301 marker_26692
marker_44072 marker_40123
marker_38241 marker_38866
marker_39125
marker_38761
marker_38741
marker_42586
marker_45143
marker_40312
marker_44784
marker_40921 marker_38240
marker_31549 marker_37364
marker_40618
marker_40605 marker_42120
marker_43224 marker_32816
marker_34374 marker_45304
marker_45452
marker_33237
marker_44231
marker_42136
marker_38488 marker_39837
marker_32386 marker_19990
marker_32345
marker_39972
marker_24983 marker_28504
marker_44632
marker_28592 marker_43366
marker_43238
marker_37523
marker_44750
marker_40992
marker_32628 marker_44587
marker_39461
marker_22932
marker_15311
marker_36508 marker_39848
marker_30046
marker_31698 marker_35289
marker_29248 marker_43167
marker_45221 marker_41189
marker_44399 marker_27995
marker_1642 marker_39381
marker_44514 marker_37235
marker_44314 marker_29488
marker_34474 marker_38185
marker_41789
marker_27310 marker_37992
marker_15712
marker_18864
marker_36745

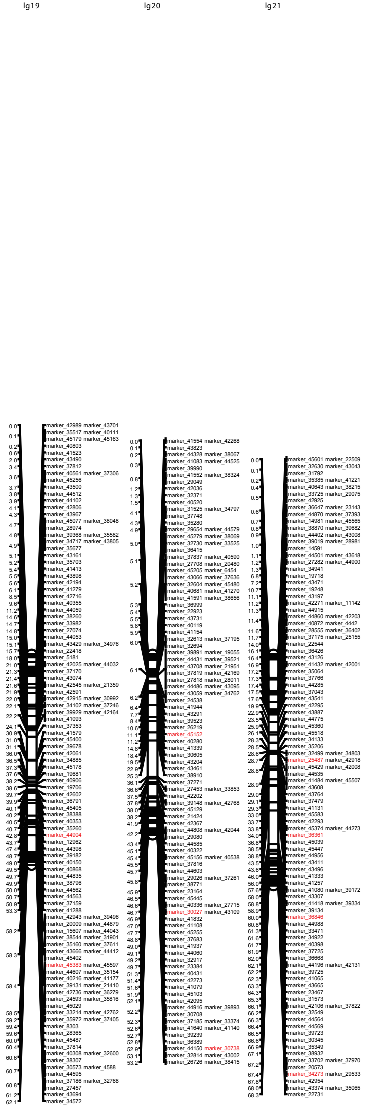

lg19
0.0
0.1
0.2
0.6
2.0
3.4
3.6
3.7
3.8
3.9
4.1
4.3
4.7
4.8
4.9
5.1
5.2
5.4
5.5
5.6
6.1
8.5
9.6
11.2
14.6
14.7
14.8
15.0
15.1
15.7
18.0
21.0
21.3
21.4
21.6
21.9
22.0
22.1
22.2
24.1
30.9
31.0
31.1
36.0
36.5
37.3
37.6
38.2
38.6
39.7
39.9
40.1
40.2
40.5
40.7
42.8
43.7
47.4
48.7
49.0
49.5
49.7
49.9
50.0
50.7
50.9
53.3
58.2
58.3
58.4
58.5
59.2
59.4
59.6
60.0
60.4
60.6
60.7
60.8
61.2
62.1
marker_42989 marker_43701
marker_35517 marker_40111
marker_45179 marker_45163
marker_40803
marker_41523
marker_43490
marker_37812
marker_40561 marker_37306
marker_45256
marker_43500
marker_44512
marker_44102
marker_42806
marker_43967
marker_45077 marker_38048
marker_28974
marker_39368 marker_35582
marker_34717 marker_43805
marker_35677
marker_43161
marker_35703
marker_41413
marker_43898
marker_42194
marker_41279
marker_42716
marker_40355
marker_44059
marker_38260
marker_33982
marker_27074
marker_44053
marker_43429 marker_34976
marker_22418
marker_5181
marker_42025 marker_44032
marker_37170
marker_43074
marker_42545 marker_21359
marker_42591
marker_42915 marker_30992
marker_34102 marker_37246
marker_39929 marker_42164
marker_41093
marker_37353
marker_41579
marker_45400
marker_39678
marker_42061
marker_34885
marker_45178
marker_19681
marker_40906
marker_19706
marker_42602
marker_36791
marker_45405
marker_38388
marker_40353
marker_35260
marker_44904
marker_12962
marker_44398
marker_39182
marker_40150
marker_40868
marker_44835
marker_38796
marker_44562
marker_44563
marker_37159
marker_41288
marker_42943 marker_39496
marker_20009 marker_44879
marker_15607 marker_44043
marker_38544 marker_31901
marker_35160 marker_37611
marker_43666 marker_44412
marker_45402
marker_45383 marker_45597
marker_44607 marker_35154
marker_40216 marker_41177
marker_39131 marker_21410
marker_42736 marker_36279
marker_24593 marker_35816
marker_45029
marker_33214 marker_42762
marker_35972 marker_37405
marker_8303
marker_28365
marker_45487
marker_37814
marker_40308 marker_32600
marker_38307
marker_30573 marker_4588
marker_44595
marker_37186 marker_32768
marker_27457
marker_43694
marker_34572
lg20
0.0
0.1
0.2
0.3
0.8
1.2
1.3
1.5
4.1
4.3
4.9
5.0
5.1
5.2
5.3
5.4
5.5
5.8
5.9
6.0
6.1
6.2
6.4
7.7
8.4
10.6
11.1
11.2
14.8
18.4
19.5
22.9
25.3
36.1
36.6
37.5
37.8
38.0
38.2
41.9
42.2
43.4
45.1
45.4
45.5
45.7
45.8
45.9
46.5
46.6
46.7
46.9
47.0
47.7
48.8
49.0
49.1
49.2
49.7
51.3
51.4
51.6
51.9
52.1
52.2
52.3
52.6
52.7
52.8
52.9
53.1
53.2
marker_41554 marker_42268
marker_43823
marker_44328 marker_38067
marker_41083 marker_44525
marker_39990
marker_41552 marker_38324
marker_29049
marker_42036
marker_32371
marker_40520
marker_31525 marker_34797
marker_37748
marker_35280
marker_29654 marker_44579
marker_45279 marker_38069
marker_32730 marker_33525
marker_36415
marker_37837 marker_40590
marker_27708 marker_20480
marker_45205 marker_6454
marker_43066 marker_37636
marker_32604 marker_45480
marker_40681 marker_41270
marker_41591 marker_38656
marker_36999
marker_22923
marker_43731
marker_40119
marker_41154
marker_32613 marker_37195
marker_32694
marker_39891 marker_19055
marker_44431 marker_39521
marker_43708 marker_21951
marker_37819 marker_42169
marker_27818 marker_28011
marker_44486 marker_43095
marker_43059 marker_34762
marker_24538
marker_41944
marker_43291
marker_39523
marker_26219
marker_45152
marker_40280
marker_41339
marker_30605
marker_43204
marker_43461
marker_38910
marker_37271
marker_27453 marker_33853
marker_42202
marker_39148 marker_42768
marker_45129
marker_21424
marker_42367
marker_44808 marker_42044
marker_29080
marker_44585
marker_40322
marker_45156 marker_40538
marker_37816
marker_44603
marker_29026 marker_37261
marker_38771
marker_23164
marker_45445
marker_40336 marker_27715
marker_30027 marker_43109
marker_41832
marker_41108
marker_45255
marker_37683
marker_41937
marker_44060
marker_32917
marker_23384
marker_40431
marker_42273
marker_41079
marker_45103
marker_42095
marker_44916 marker_39893
marker_30708
marker_37185 marker_33374
marker_41640 marker_41140
marker_39239
marker_36389
marker_44150 marker_30738
marker_32814 marker_43002
marker_26726 marker_38415
lg21
0.0
0.1
0.2
0.4
0.5
0.6
0.7
0.8
0.9
1.0
1.1
1.2
1.3
6.8
7.2
10.7
11.1
11.2
11.3
11.4
11.6
11.7
14.0
16.1
16.4
16.9
17.2
17.3
17.4
17.5
17.6
19.9
22.9
23.5
25.9
26.1
28.3
28.5
28.6
28.7
28.8
28.9
29.0
29.1
29.3
33.0
33.7
33.8
34.0
36.8
38.5
38.8
43.5
43.6
46.0
56.0
57.6
58.0
58.8
58.9
60.0
60.8
61.3
61.6
61.7
61.9
62.0
62.1
62.2
62.3
63.3
64.3
65.3
66.1
66.2
66.3
66.4
66.5
66.6
66.9
67.1
67.2
67.4
67.8
68.0
68.3
marker_45601 marker_22509
marker_32630 marker_43043
marker_31792
marker_35385 marker_41221
marker_40643 marker_38215
marker_33725 marker_29075
marker_42925
marker_36647 marker_23143
marker_44870 marker_37393
marker_14981 marker_45565
marker_38870 marker_39682
marker_44402 marker_43008
marker_39019 marker_28981
marker_14591
marker_44501 marker_43618
marker_27282 marker_44900
marker_34941
marker_19718
marker_43471
marker_19248
marker_43197
marker_42271 marker_11142
marker_44915
marker_44860 marker_42203
marker_40872 marker_4442
marker_28555 marker_36402
marker_37175 marker_25155
marker_22544
marker_36426
marker_43126
marker_41432 marker_42001
marker_35064
marker_37766
marker_44285
marker_37043
marker_43541
marker_42295
marker_43887
marker_44775
marker_45360
marker_45518
marker_34133
marker_35206
marker_32499 marker_34803
marker_25487 marker_42918
marker_45429 marker_42008
marker_44535
marker_41484 marker_45507
marker_43608
marker_43764
marker_37479
marker_41131
marker_45583
marker_42293
marker_45374 marker_44273
marker_36361
marker_45039
marker_45447
marker_44956
marker_43411
marker_43496
marker_41333
marker_41257
marker_41080 marker_39172
marker_43307
marker_41418 marker_39334
marker_39134
marker_36846
marker_44988
marker_33471
marker_34922
marker_40398
marker_37725
marker_36668
marker_44196 marker_42131
marker_39725
marker_41065
marker_43665
marker_23467
marker_31573
marker_42106 marker_37822
marker_32549
marker_44564
marker_44569
marker_39723
marker_30345
marker_35349
marker_38932
marker_33702 marker_37970
marker_20573
marker_34273 marker_29533
marker_42954
marker_43374 marker_35065
marker_22731

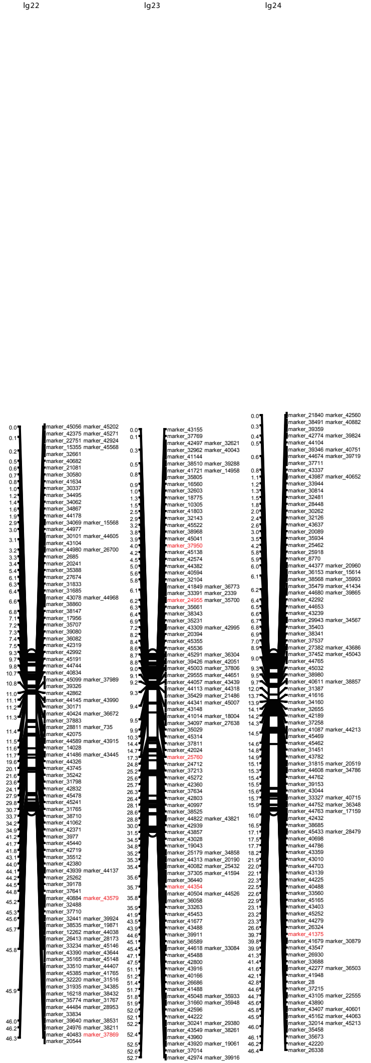
lg22
lg23
lg24
0.0
0.1
0.2
0.5
0.6
0.7
0.8
1.0
1.2
1.4
1.6
1.9
2.9
3.0
3.1
3.2
3.3
3.4
5.4
6.1
6.3
6.4
6.6
6.8
7.1
7.2
7.3
7.4
7.5
9.3
9.7
9.8
10.7
10.8
11.0
11.1
11.2
11.3
11.4
11.5
11.6
11.7
19.6
20.1
21.6
23.6
24.1
27.9
29.8
30.7
33.7
34.2
34.9
41.2
41.7
41.8
43.1
44.0
44.1
44.2
44.5
44.8
45.2
45.3
45.6
45.7
45.8
45.9
46.0
46.2
46.3
marker_45056 marker_45202
marker_42375 marker_45271
marker_22751 marker_42924
marker_15355 marker_45568
marker_32661
marker_40682
marker_21081
marker_30580
marker_41634
marker_30337
marker_34495
marker_34062
marker_34867
marker_44178
marker_34069 marker_15568
marker_44977
marker_30101 marker_44605
marker_43104
marker_44980 marker_26700
marker_2685
marker_20241
marker_35388
marker_27674
marker_31833
marker_31685
marker_43078 marker_44968
marker_38860
marker_38147
marker_17956
marker_35707
marker_39080
marker_36082
marker_42319
marker_42992
marker_45191
marker_44744
marker_40834
marker_45099 marker_37989
marker_39326
marker_42862
marker_44145 marker_43990
marker_30171
marker_40424 marker_36672
marker_37883
marker_28811 marker_735
marker_42075
marker_44589 marker_43915
marker_14028
marker_41486 marker_43445
marker_44326
marker_43745
marker_35242
marker_31798
marker_42832
marker_45478
marker_45241
marker_31765
marker_38710
marker_41062
marker_42371
marker_3977
marker_45440
marker_42719
marker_35512
marker_42380
marker_43939 marker_44137
marker_25262
marker_39178
marker_37641
marker_40884 marker_43579
marker_32488
marker_37710
marker_32441 marker_39924
marker_38535 marker_19871
marker_12262 marker_44038
marker_26413 marker_28173
marker_33234 marker_45146
marker_43390 marker_43644
marker_35165 marker_45148
marker_33510 marker_44407
marker_45385 marker_41765
marker_32220 marker_31516
marker_31935 marker_34385
marker_16218 marker_38432
marker_35774 marker_31767
marker_44484 marker_28953
marker_33834
marker_39640 marker_38531
marker_24976 marker_38211
marker_40483 marker_37869
marker_20544
0.0
0.1
0.3
0.5
0.8
0.9
1.0
1.3
1.5
2.5
2.9
3.2
3.8
3.9
4.0
4.2
4.5
5.0
5.6
5.8
6.1
6.2
6.3
6.4
6.9
7.2
8.2
8.4
8.6
8.7
8.8
9.0
9.1
9.2
9.3
9.4
9.5
9.9
10.3
14.4
14.7
17.3
24.8
25.2
25.6
25.7
26.3
26.4
28.1
28.6
28.8
30.0
31.5
34.5
34.8
35.2
35.3
35.4
35.6
35.7
35.8
35.9
41.9
43.5
43.9
44.6
45.1
45.4
47.1
47.5
51.1
51.2
51.4
51.7
51.8
51.9
52.0
52.1
52.2
52.4
52.5
52.6
52.7
marker_43155
marker_37769
marker_42497 marker_32621
marker_32962 marker_40043
marker_41144
marker_38510 marker_39288
marker_41721 marker_14958
marker_35805
marker_16560
marker_32603
marker_18775
marker_10305
marker_41803
marker_32143
marker_45522
marker_38968
marker_45041
marker_37950
marker_45138
marker_42574
marker_44382
marker_40594
marker_32104
marker_41849 marker_36773
marker_33391 marker_2339
marker_24955 marker_35700
marker_35661
marker_38343
marker_35231
marker_43309 marker_42995
marker_20394
marker_45355
marker_45536
marker_45291 marker_36304
marker_39426 marker_42051
marker_45003 marker_37806
marker_29555 marker_44651
marker_44057 marker_43439
marker_44113 marker_44318
marker_35429 marker_21486
marker_44341 marker_45007
marker_43148
marker_41014 marker_18004
marker_34097 marker_27638
marker_35029
marker_45314
marker_37811
marker_42024
marker_25760
marker_24712
marker_37213
marker_45272
marker_42360
marker_37634
marker_42803
marker_40997
marker_38525
marker_44822 marker_43821
marker_42939
marker_43857
marker_43028
marker_19043
marker_25179 marker_34858
marker_44313 marker_20190
marker_40082 marker_25432
marker_37305 marker_41594
marker_36440
marker_44354
marker_40504 marker_44526
marker_36058
marker_33263
marker_45453
marker_41677
marker_43488
marker_39911
marker_36589
marker_44618 marker_33084
marker_45488
marker_42800
marker_43916
marker_40166
marker_26686
marker_41488
marker_45048 marker_35933
marker_31660 marker_35948
marker_42596
marker_44222
marker_30241 marker_29380
marker_43549 marker_38261
marker_43960
marker_43920 marker_19061
marker_37014
marker_42974 marker_39916
0.0
0.3
0.4
0.5
0.6
0.8
1.1
1.2
1.3
1.5
1.8
2.0
2.4
2.6
3.0
3.5
4.2
5.8
5.9
6.0
6.1
6.2
6.4
6.5
6.6
6.7
6.8
6.9
7.0
8.9
9.0
9.3
9.5
9.7
12.0
13.7
13.9
14.1
14.2
14.3
14.5
14.6
14.8
14.9
15.1
15.3
15.4
15.5
15.6
15.7
15.9
16.0
16.5
17.1
17.5
17.7
18.2
21.9
22.0
22.1
22.3
22.5
22.6
22.9
23.1
23.2
23.3
26.6
39.7
39.8
39.9
41.3
41.4
41.6
42.1
42.8
44.6
44.7
45.6
45.8
45.9
46.0
46.1
46.2
46.4
marker_21840 marker_42560
marker_38491 marker_40882
marker_39359
marker_42774 marker_39824
marker_44104
marker_39346 marker_40751
marker_44674 marker_39719
marker_37711
marker_43337
marker_43987 marker_40652
marker_33944
marker_30814
marker_32481
marker_28448
marker_30262
marker_32126
marker_43637
marker_20089
marker_35934
marker_25462
marker_25918
marker_8770
marker_44377 marker_20960
marker_36153 marker_15614
marker_38568 marker_35993
marker_35479 marker_41434
marker_44680 marker_39865
marker_42292
marker_44653
marker_43239
marker_29943 marker_34567
marker_35403
marker_38341
marker_37537
marker_27382 marker_43686
marker_37452 marker_45043
marker_44765
marker_45032
marker_38980
marker_40611 marker_38857
marker_31387
marker_41616
marker_34160
marker_32655
marker_42189
marker_37258
marker_41087 marker_44213
marker_45469
marker_45462
marker_31451
marker_43782
marker_31815 marker_20519
marker_44608 marker_34786
marker_44762
marker_39153
marker_43044
marker_33327 marker_40715
marker_44752 marker_36348
marker_44763 marker_17159
marker_42432
marker_38685
marker_45433 marker_28479
marker_40698
marker_44786
marker_43359
marker_43010
marker_44703
marker_43139
marker_44225
marker_40488
marker_33560
marker_45165
marker_43403
marker_45252
marker_44279
marker_26324
marker_41375
marker_41679 marker_30879
marker_43547
marker_26930
marker_33688
marker_42277 marker_36503
marker_41948
marker_28
marker_37215
marker_43105 marker_22555
marker_43890
marker_43407 marker_40601
marker_45162 marker_44063
marker_32014 marker_45213
marker_35458
marker_35673
marker_42220
marker_26338

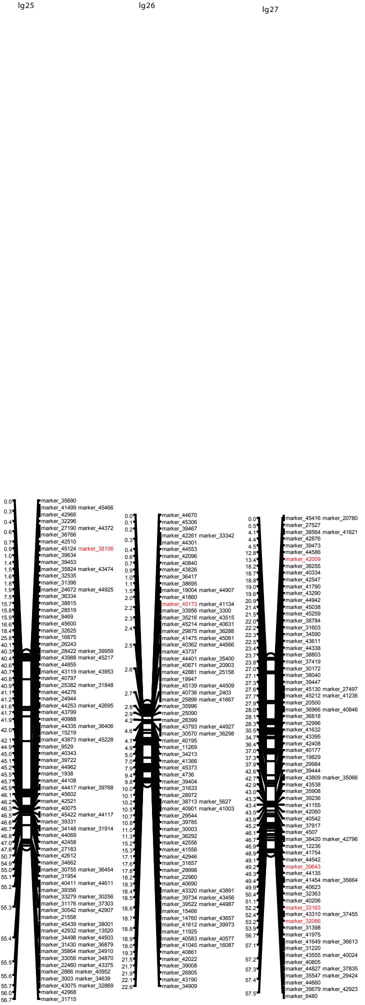
lg25
0.0
0.3
0.4
0.6
0.7
0.9
1.0
1.4
1.5
1.6
1.8
1.9
7.5
15.7
15.8
15.9
16.6
18.4
25.8
40.1
40.3
40.4
40.6
40.7
40.8
40.9
41.1
41.2
41.6
41.7
41.9
42.0
42.1
44.9
45.0
45.1
45.2
45.5
45.7
45.9
46.1
46.2
46.3
46.5
46.6
46.7
46.8
47.0
47.6
50.7
54.9
55.0
55.1
55.2
55.3
55.4
55.5
55.6
55.7
56.0
56.7
marker_35690
marker_41499 marker_45466
marker_42966
marker_32296
marker_27190 marker_44372
marker_36766
marker_42510
marker_45124 marker_38106
marker_39634
marker_39453
marker_35824 marker_43474
marker_32535
marker_31396
marker_24672 marker_44925
marker_36334
marker_38815
marker_28519
marker_9469
marker_45600
marker_32625
marker_16875
marker_26243
marker_28422 marker_39959
marker_43988 marker_45217
marker_44855
marker_43119 marker_43953
marker_40797
marker_25382 marker_31848
marker_44276
marker_24944
marker_44253 marker_42695
marker_43799
marker_40988
marker_44335 marker_36406
marker_15219
marker_43873 marker_45228
marker_9529
marker_40343
marker_39722
marker_44962
marker_1938
marker_44108
marker_44417 marker_39768
marker_45602
marker_42521
marker_40075
marker_45422 marker_44117
marker_39331
marker_34148 marker_31914
marker_44069
marker_42458
marker_27183
marker_42612
marker_34662
marker_30755 marker_36454
marker_31954
marker_40411 marker_44611
marker_39356
marker_33279 marker_30256
marker_31176 marker_37303
marker_30542 marker_42907
marker_21558
marker_45439 marker_38001
marker_42932 marker_13520
marker_34498 marker_44503
marker_31430 marker_36879
marker_35864 marker_24910
marker_33056 marker_34870
marker_22460 marker_43375
marker_2866 marker_40952
marker_3003 marker_34639
marker_43075 marker_32869
marker_42968
marker_31715
lg26
0.0
0.1
0.2
0.3
0.4
0.6
0.7
0.9
1.0
1.1
1.5
2.0
2.2
2.3
2.4
2.5
2.6
2.7
2.8
2.9
4.2
4.6
4.7
4.9
5.0
7.0
7.9
9.4
9.8
10.0
10.1
10.2
10.5
10.7
10.8
11.0
11.3
15.2
15.3
17.1
17.6
17.8
18.2
18.3
18.4
18.5
18.6
18.7
18.8
18.9
19.0
19.3
21.5
21.7
21.9
22.1
22.6
marker_44670
marker_45306
marker_39467
marker_42261 marker_33342
marker_44301
marker_44553
marker_42096
marker_40840
marker_43826
marker_36417
marker_38695
marker_19004 marker_44907
marker_41860
marker_45173 marker_41134
marker_33956 marker_3300
marker_35216 marker_43515
marker_45214 marker_40631
marker_29875 marker_36288
marker_41475 marker_45061
marker_40362 marker_44566
marker_43737
marker_44401 marker_35400
marker_40671 marker_20903
marker_42881 marker_25158
marker_19947
marker_45139 marker_44509
marker_40736 marker_2403
marker_25899 marker_41667
marker_35996
marker_25090
marker_28399
marker_43793 marker_44927
marker_30570 marker_36298
marker_40195
marker_11269
marker_34213
marker_41366
marker_45373
marker_4736
marker_39404
marker_31633
marker_28972
marker_38713 marker_5627
marker_40901 marker_41003
marker_29544
marker_39785
marker_30003
marker_36292
marker_42556
marker_41556
marker_42946
marker_31657
marker_29998
marker_22960
marker_40690
marker_43320 marker_43891
marker_39734 marker_43456
marker_39522 marker_44987
marker_15466
marker_14760 marker_43657
marker_41612 marker_39973
marker_11925
marker_40583 marker_40577
marker_41045 marker_16087
marker_40861
marker_42022
marker_39008
marker_26805
marker_43190
marker_34909
lg27
0.0
0.5
4.1
4.4
4.5
12.8
13.4
18.2
18.7
18.8
19.0
19.6
20.9
21.4
21.5
22.0
22.2
22.3
22.5
23.4
23.7
23.8
27.0
27.1
27.3
27.6
27.7
27.9
28.0
28.1
28.3
30.5
34.7
36.4
37.0
37.3
37.9
42.6
42.7
42.9
43.0
43.4
43.5
43.9
45.2
46.1
46.7
46.9
48.9
49.1
49.2
49.3
49.4
50.4
51.1
52.2
52.4
53.2
53.9
56.7
57.1
57.2
57.3
57.4
57.5
marker_45416 marker_20780
marker_27527
marker_38564 marker_41621
marker_42876
marker_39473
marker_44586
marker_42009
marker_38255
marker_40334
marker_42547
marker_41790
marker_43290
marker_44942
marker_45038
marker_45259
marker_38784
marker_31603
marker_34590
marker_43611
marker_44338
marker_38803
marker_37419
marker_30172
marker_38040
marker_39447
marker_45130 marker_27497
marker_45212 marker_41236
marker_20500
marker_36966 marker_40846
marker_36818
marker_32996
marker_41632
marker_43395
marker_42408
marker_40177
marker_19829
marker_29984
marker_39444
marker_43809 marker_35066
marker_43538
marker_35908
marker_39236
marker_41155
marker_42060
marker_40542
marker_37917
marker_4507
marker_38420 marker_42796
marker_12236
marker_41754
marker_44542
marker_39643
marker_44135
marker_41454 marker_35664
marker_40623
marker_32363
marker_40206
marker_33163
marker_43310 marker_37455
marker_32086
marker_31398
marker_41975
marker_41649 marker_36613
marker_31220
marker_43555 marker_40024
marker_40805
marker_44827 marker_37835
marker_35547 marker_29424
marker_44660
marker_35679 marker_42923
marker_6480

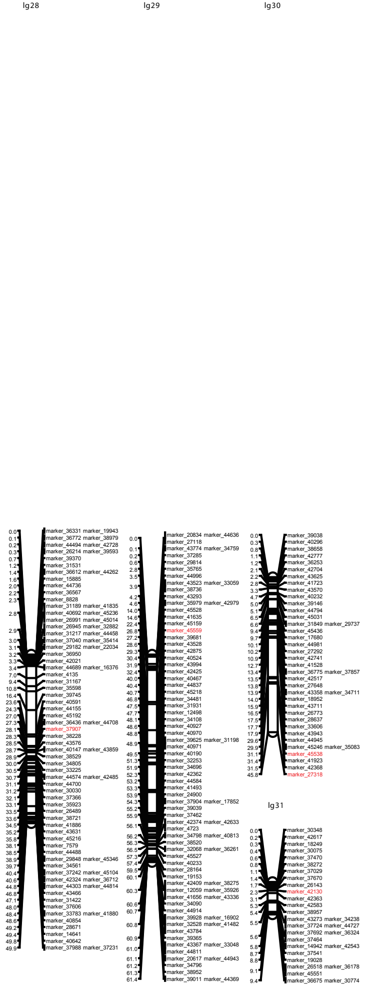
lg28
lg29
lg30
0.0 marker_36331 marker_19943
0.1 marker_36772 marker_38979
0.2 marker_44494 marker_42728
0.3 marker_26214 marker_39593
0.7 marker_39370
1.2 marker_31531
1.4 marker_36612 marker_44262
1.6 marker_15885
2.0 marker_44736
2.2 marker_36567
2.3 marker_8828
2.8 marker_31189 marker_41835
marker_40692 marker_45236
marker_26991 marker_45014
marker_26945 marker_32882
2.9 marker_31217 marker_44458
3.0 marker_37040 marker_35414
3.1 marker_29182 marker_22034
3.2 marker_36950
3.3 marker_42021
3.4 marker_44689 marker_16376
7.0 marker_4135
9.4 marker_31167
10.8 marker_35598
16.4 marker_39745
23.6 marker_40591
24.3 marker_44155
27.0 marker_45192
27.3 marker_36436 marker_44708
28.1 marker_37907
28.3 marker_38228
28.5 marker_43576
28.7 marker_40147 marker_43859
28.9 marker_38529
30.0 marker_34805
30.5 marker_33225
30.7 marker_44574 marker_42485
31.1 marker_44700
31.2 marker_30030
32.1 marker_37366
33.1 marker_35923
33.5 marker_26489
33.6 marker_38721
34.5 marker_41886
35.2 marker_43631
35.8 marker_45216
38.1 marker_7579
38.5 marker_44488
38.9 marker_29848 marker_45346
39.7 marker_34561
40.4 marker_37242 marker_45104
40.6 marker_42324 marker_36712
44.8 marker_44303 marker_44814
46.8 marker_43466
47.1 marker_31422
48.0 marker_37606
48.4 marker_33783 marker_41880
48.6 marker_40854
49.2 marker_28671
49.4 marker_14641
49.8 marker_40642
49.9 marker_37988 marker_37231
marker_20834 marker_44636
0.0 marker_27118
0.1 marker_43774 marker_34759
0.2 marker_37285
0.6 marker_29814
2.8 marker_35765
3.5 marker_44996
3.9 marker_43523 marker_33059
marker_38736
4.2 marker_43293
4.6 marker_35979 marker_42979
14.0 marker_45528
14.6 marker_41635
22.4 marker_45159
26.8 marker_45559
27.2 marker_39681
28.6 marker_43528
29.3 marker_42875
30.4 marker_40524
31.9 marker_43994
32.4 marker_42425
40.0 marker_40467
40.4 marker_44837
40.7 marker_45218
46.8 marker_34481
47.5 marker_31931
47.7 marker_12498
48.1 marker_34108
48.6 marker_40927
48.8 marker_40970
48.9 marker_39625 marker_31198
marker_40971
49.5 marker_40190
51.3 marker_32253
51.9 marker_34696
52.3 marker_42362
53.2 marker_44584
53.3 marker_41493
53.9 marker_24900
54.3 marker_37904 marker_17852
55.2 marker_39039
55.9 marker_37462
56.1 marker_42374 marker_42633
marker_4723
56.2 marker_34798 marker_40813
56.3 marker_38520
56.5 marker_32068 marker_36261
57.3 marker_45527
57.4 marker_40233
59.5 marker_28164
60.1 marker_19153
60.3 marker_42409 marker_38275
marker_12059 marker_35926
marker_41656 marker_43336
60.6 marker_34090
60.7 marker_44914
60.8 marker_39928 marker_16902
marker_32528 marker_41482
marker_43784
60.9 marker_39365
61.0 marker_43367 marker_33048
marker_44811
61.1 marker_20617 marker_44943
61.2 marker_34796
61.3 marker_38952
61.4 marker_39011 marker_44369
0.0 marker_39038
0.3 marker_40296
0.8 marker_38658
1.1 marker_42777
1.2 marker_36253
2.1 marker_42704
2.2 marker_43625
2.8 marker_41723
3.3 marker_43570
4.7 marker_40232
5.0 marker_39146
5.1 marker_44794
6.5 marker_45031
6.6 marker_31849 marker_29737
9.4 marker_45436
9.7 marker_17680
10.1 marker_44981
10.2 marker_27292
10.9 marker_42741
12.7 marker_41528
13.4 marker_36775 marker_37857
13.5 marker_42517
13.8 marker_27648
13.9 marker_43358 marker_34711
14.0 marker_18952
15.9 marker_43711
16.5 marker_26773
17.5 marker_28637
17.7 marker_33606
17.9 marker_43943
29.6 marker_44945
29.9 marker_45246 marker_35083
31.1 marker_45538
31.4 marker_41923
31.5 marker_42368
45.8 marker_27318
lg31
0.0 marker_30348
0.2 marker_42617
0.3 marker_18249
0.4 marker_30075
0.6 marker_37470
0.8 marker_38272
1.1 marker_37029
1.4 marker_37670
1.7 marker_26143
2.3 marker_42130
3.1 marker_42363
4.2 marker_42583
5.3 marker_38957
5.4 marker_43273 marker_34238
5.5 marker_37724 marker_44727
marker_37692 marker_36324
5.6 marker_37464
5.8 marker_14942 marker_42543
8.7 marker_37541
8.8 marker_19028
9.1 marker_26518 marker_36178
marker_45551
9.4 marker_36675 marker_30774
