# Supplementary figures and images for: Construction of a high density SNP linkage map of kelp (Saccharina japonica) by sequencing Taq I site associated DNA and mapping of a sex determining locus
Source: BMC Genomics. 2015 Mar 15;16(1):189. doi: 10.1186/s12864-015-1371-1 (PMC4369078; doi:10.1186/s12864-015-1371-1)

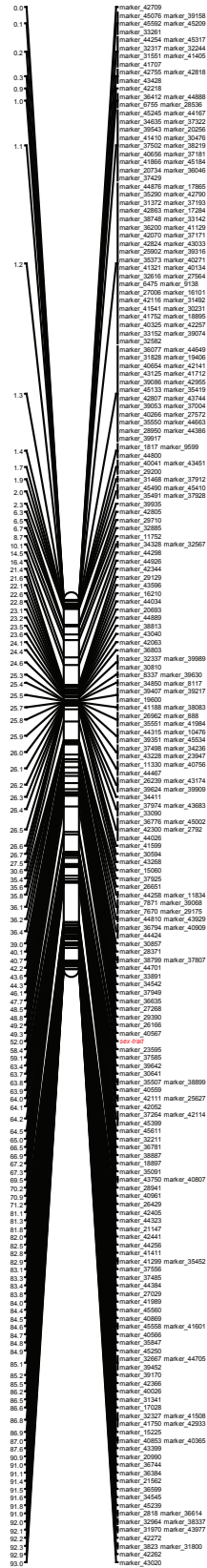

Supplement: Additional file 6: — Graphical position of the sex determining locus of kelp gametophytes. The illustrated include the sex determining locus (in red color) and its relationship to the adjacent markers on linkage group 2. [file 12864_2015_1371_MOESM6_ESM.pdf]
